# Predicting the spatial expansion of an animal population with presence‐only data

**DOI:** 10.1002/ece3.10778

**Published:** 2023-11-27

**Authors:** Owain Barton, John R. Healey, Line S. Cordes, Andrew J. Davies, Graeme Shannon

**Affiliations:** ^1^ School of Natural Sciences Bangor University Bangor UK; ^2^ Norwegian Institute for Nature Research Trondheim Norway; ^3^ Department of Biological Sciences University of Rhode Island Kingston Rhode Island USA

**Keywords:** *Capreolus capreolus*, hybrid model, mechanistic, population management, presence‐only data, range expansion, spatially explicit spread, wildlife management

## Abstract

Predictive models can improve the efficiency of wildlife management by guiding actions at the local, landscape and regional scales. In recent decades, a vast range of modelling techniques have been developed to predict species distributions and patterns of population spread. However, data limitations often constrain the precision and biological realism of models, which make them less useful for supporting decision‐making. Complex models can also be challenging to evaluate, and the results are often difficult to interpret for wildlife management practitioners. There is therefore a need to develop techniques that are appropriately robust, but also accessible to a range of end users. We developed a hybrid species distribution model that utilises commonly available presence‐only distribution data and minimal demographic information to predict the spread of roe deer (*Capreolus caprelous*) in Great Britain. We take a novel approach to representing the environment in the model by constraining the size of habitat patches to the home‐range area of an individual. Population dynamics are then simplified to a set of generic rules describing patch occupancy. The model is constructed and evaluated using data from a populated region (England and Scotland) and applied to predict regional‐scale patterns of spread in a novel region (Wales). It is used to forecast the relative timing of colonisation events and identify important areas for targeted surveillance and management. The study demonstrates the utility of presence‐only data for predicting the spread of animal species and describes a method of reducing model complexity while retaining important environmental detail and biological realism. Our modelling approach provides a much‐needed opportunity for users without specialist expertise in computer coding to leverage limited data and make robust, easily interpretable predictions of spread to inform proactive population management.

## Graphical abstract

This study developed a hybrid species distribution model using presence‐only distribution data and minimal demographic information to predict the spread of roe deer in Great Britain. The model utilised a novel approach to representing the environment by constraining the size of habitat patches to the home‐range area of an individual, simplifying population dynamics to a set of generic rules. The model was evaluated and applied to predict regional‐scale patterns of spread in a novel region, demonstrating the utility of presence‐only data for predicting animal species' spread while retaining environmental detail and biological realism.

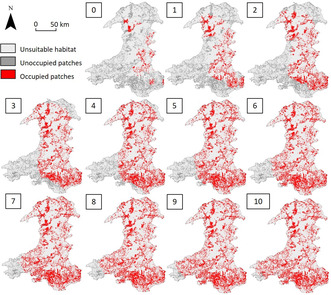

## 1 INTRODUCTION

Understanding how characteristics of the environment influence species distributions is a fundamental aim of spatial ecology (Elith & Leathwick, 2009; Skidmore et al., 2011). Many terrestrial animal populations have altered their geographic ranges in response to human activities (e.g. habitat modification, Wilson, Davies, et al., 2009; Wilson, Dormontt, et al., 2009) and anthropogenic climate change (e.g. Dawe & Boutin, 2016). Shifts in animal distributions lead to novel biotic and abiotic interactions that may affect ecosystem health and functioning (Pacifici et al., 2020; Pessarrodona et al., 2019). Forecasting changes in species distributions and predicting the relative timing of colonisation events is therefore essential for effective conservation planning (Aben et al., 2016; Battini et al., 2019; Fordham et al., 2013). Reliable predictions can be used to distribute resources for surveillance and management efficiently to vulnerable habitats and landscape features that benefit expansion (e.g. habitat corridors, Akashi et al., 2016; Bottrill et al., 2008; Tilman et al., 2017).

Species–environment relationships are commonly investigated using correlative species distribution models, which empirically relate species distributions to environmental variables, such as precipitation or land use (Elith & Leathwick, 2009). For range‐expanding species, correlative models can provide robust predictions of the spatial distribution of suitable habitats in novel areas (Elith et al., 2010; Lake et al., 2020). However, the probability and timing of population spread are likely to be influenced by a range of other factors, such as demography, physiology, dispersal and species interactions (Dormann et al., 2012). Mechanistic models can be used to simulate these underlying ecological processes and investigate the functional relationships between them and species distributions (Kearney & Porter, 2009; McLane et al., 2011; Wallentin, 2017). Combining correlative and mechanistic models (i.e. as in ‘coupled’ or ‘hybrid’ models, ‘hybrid’ models hereafter) improves the realism of predictions and offers a powerful tool for predicting changes in distribution over time (Buckley et al., 2010; Dormann et al., 2012; Fordham et al., 2013). Hybrid models can be implemented using a range of tools, such as MigClim (Engler et al., 2012), KISSMig (Nobis & Normand, 2014) and demoniche (Nenzén et al., 2012). Typically, the output from a correlative model (e.g. a raster map) is used to represent the environment in simulations of population dynamics and dispersal. This allows key parameters of simulations (e.g. local carrying capacity) to be constrained by features of the modelled environment (e.g. habitat suitability, Dormann et al., 2012; Singer et al., 2018).

Environmental representation and model structure are important factors that influence the realism, data requirements and complexity of hybrid models. Simulations are often based on the representation of species as automata that populate a raster grid of regular cells (‘grid‐based’ models hereafter, Keshtkar & Voigt, 2016; Louca et al., 2015). Grid‐based models are conceptually simple, generally require minimal data to parameterise and are computationally efficient to implement (Bian, 2003; McLane et al., 2011). Although logistically convenient, they are usually best suited to modelling plant species (Aben et al., 2016; Bian, 2007; Vuilleumier & Metzger, 2006). The fixed cell size of the raster grid implies that ecological processes, such as survival, reproduction, emigration and dispersal, occur at the same scale, which is unrealistic for most animal species (Bocedi, Zurell, et al., 2014; Vuilleumier & Metzger, 2006; Wallentin, 2017). Representing the environment as continuous space is also unsuitable for species that show a preference for discrete habitat features (e.g. woodlands, Bian, 2003; McLane et al., 2011).

Alternatively, landscapes may be represented as a network of patches (‘patch‐based’ models hereafter). Generalising the continuous raster grid produced by a correlative model into a landscape of patches typically requires the application of a suitability threshold. Neighbouring cells with suitability values at or above this threshold are then aggregated to delineate discrete patches of suitable habitat embedded in a matrix of less hospitable environments (Berec, 2002; Bian, 2003). Patch‐based models therefore offer a more realistic representation of the environment as the units of the landscape (patches) reflect the geometry, distribution and composition of natural features (Holland et al., 2007; Vuilleumier & Metzger, 2006). Patches also facilitate modelling at multiple spatial scales. For example, fine‐scale movement between patches during dispersal may be simulated as a correlated random walk (e.g. Bocedi, Zurell, et al., 2014) using high‐resolution raster maps. Population dynamics may be simulated at the local scale of the patch and patterns of population spread emerge at the landscape or regional scale (Austin & Van Niel, 2011; Bocedi, Palmer, et al., 2014; Wallentin, 2017). However, the requirements of modelling population dynamics can affect how patch‐based landscapes are represented, as patches typically need to be large enough to accommodate multiple individuals (Berec, 2002; Cavanaugh et al., 2014). Applying a size threshold eliminates patches that are unable to sustain a sub‐population, but these may form a network of suitable habitats that contributes to the viability and spread of the total population (Fahrig, 2020; Tulloch, Barnes, et al., 2016). Therefore, inaccurate representation of the environment at the landscape scale can affect model predictions at the broader regional scale (Bian, 2007; Bocedi et al., 2012).

Currently, there are limited tools available to implement conceptually simple hybrid models (e.g. KISSMig, Nobis & Normand, 2014) that utilise patch‐based environments. Parameterising hybrid models and achieving a balance between complexity and biological realism can also be challenging. Estimating patterns of colonisation and extinction through explicit simulation of population dynamics typically requires detailed demographic information, such as survival rates, fecundity, carrying capacity, emigration rates and sex ratios, which are unavailable for many species (Dormann et al., 2012; Kearney & Porter, 2009; Thuiller et al., 2013). Interpreting such complex models presents a further challenge for wildlife managers as they generally cannot be evaluated using conventional statistical methods (O'Sullivan et al., 2016; Wallentin, 2017). For practical applications, there is a need for less data‐intensive models that are biologically realistic but also simple enough to be interpreted and used effectively (Addison et al., 2013; Tulloch, Sutcliffe, et al., 2016).

The expansion of the roe deer (*Capreolus capreolus*) population in Wales, UK provides a good example of a wildlife management scenario that can be informed by predictive modelling. Although native to Britain, the numbers and geographic range of roe deer have expanded rapidly over recent decades due to reduced persecution, afforestation and the absence of natural predators (Apollonio et al., 2010; Linnell et al., 2020; Ward, 2005). While expansion may be seen as a conservation success, the potential effects of roe deer on sensitive habitats (e.g. ancient woodland) are a cause for concern (Gill & Morgan, 2010; Linnell et al., 2020). Browsing by roe deer has been shown to impede tree growth (Bergquist et al., 2009; Kay, 1993) and natural regeneration (Cutini et al., 2011; Petersson et al., 2019), reduce ground flora biodiversity (Kirkby, 2001) and quality of woodland habitat for bird species (Gill & Fuller, 2007) as well as cause damage to agricultural crops (Kjøstvedt et al., 1998; Putman, 1986). Roe deer are abundant throughout most of England and Scotland and are beginning to recolonize parts of Wales (Croft et al., 2019; Ward, 2005). Predictions of population spread in Wales are needed to guide surveillance and inform proactive mitigation efforts.

We aim to address this need by developing a hybrid species distribution model that can be parameterised and evaluated using data commonly available to wildlife management practitioners. We demonstrate our approach using opportunistically collected presence‐only distribution data for roe deer in mainland Great Britain. Records of species occurrences in a populated region (England and Scotland) are used to produce a habitat suitability map from a correlative species distribution model. This map is then generalised to represent the environment in a hybrid model as a landscape of small patches, based on the home‐range area of an individual (an ‘individual‐sized patch’). Basic demographic and dispersal information are used in simulations to predict regional‐scale patterns of population spread as a function of the size, quality and connectivity of individual‐sized patches. The hybrid model is first evaluated using observations of historical distribution change in England and Scotland and then applied to predict the population spread of roe deer in a novel region, Wales.

To be an effective tool for management, it was important that our model outputs were easily interpretable by practitioners and produced at a fine enough spatial resolution to identify potentially vulnerable landscape features (e.g. individual woodlands). Achieving temporal accuracy was considered less critical, as predicting the relative timing of colonisation events (e.g. region X is likely to be colonised before region Y) would be sufficient to set management priorities (e.g. targeted surveillance in region X). The objectives were to (1) evaluate the efficacy of representing the environment as a landscape of individual‐sized patches to predict patterns of population spread, (2) test different methods of generalising a habitat suitability map into individual‐sized patches, (3) predict the suitability of habitat and potential future range of the roe deer population in Wales and (4) predict the relative timing of colonisation events for roe deer in Wales, assuming the population realises its potential range.

## 2 METHODS

### 2.1 Study area

**FIGURE 1:**
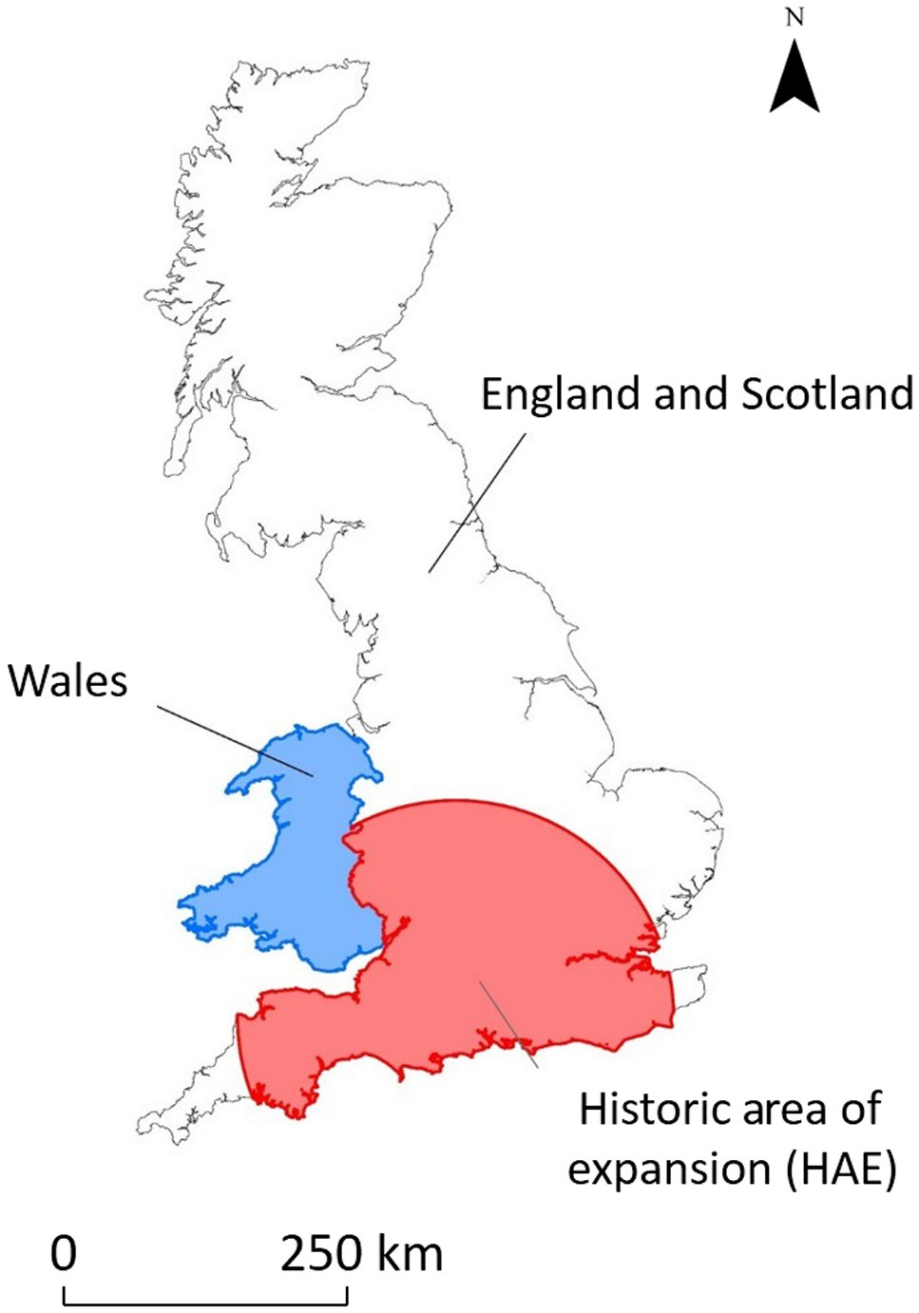
Map of the study area (mainland Great Britain) showing the boundaries of the two regions defined for the analyses and the historic area of expansion (HAE) within the England and Scotland region that was used for evaluation of the hybrid models.

The study area covered mainland Great Britain (218,819 km^2^), divided into two regions: England and Scotland (198,569 km^2^), where roe deer populations are well established and Wales (20,250 km^2^), where numbers are much lower (Figure 1). Evaluation of the hybrid models was achieved using occurrence data from an area within the England and Scotland region where the expansion of roe deer has been observed from 1960 to 2016, defined as the historic area of expansion (HAE, 60,349 km^2^, Figure 1).

### 2.2 Modelling approach

Our method consisted of five steps: (Step 1) habitat suitability was estimated from a populated region (England and Scotland) using a correlative species distribution model, (Step 2) the resultant habitat suitability map (HSM) was generalised into a landscape of individual‐sized patches to represent the environment in a hybrid model, (Step 3) demographic parameters were simplified to simulate patch occupancy for multiple time steps, (Step 4) model evaluation was performed using historic distribution data and (Step 5) the model was applied to a novel region (Wales). Predictions of population spread were based on simulations made using a mechanistic modelling platform, RangeShifter (Bocedi, Palmer, et al., 2014). RangeShifter was chosen because it is versatile, freely available and does not require any expertise in computer coding to parameterise. Furthermore, it is possible in RangeShifter to incorporate environmental information using multiple independent layers that describe: patch geometry and distribution, patch quality/composition and landscape‐associated costs of moving between patches (Bocedi, Palmer, et al., 2014). In our approach, these layers were derived from the correlative model, as described in the following sections.

#### 2.2.1 Step 1. Estimating habitat suitability

Habitat suitability was estimated using a Maximum Entropy (MaxEnt) model implemented with the ‘dismo’ package (Hijmans et al., 2017) in R (R Core Development Team, 2019). The model was trained and tested with environmental and roe deer occurrence data from the populated England and Scotland regions (MaxEnt version 3.4.0; Phillips et al., 2017).

##### Occurrence data

Data on roe deer sightings were taken for the period 1953–2016 from the National Biodiversity Network Gateway (www.nbnatlas.org) and regional wildlife trusts in Wales (Appendix S1) during December 2016. These were characteristic of presence‐only data as they were collected from a range of sources (e.g. the general public) and the sampling effort was indeterminable. Only occurrence records with a locational precision of 100 m were considered for analysis (England and Scotland, *n* = 3843). The records from Wales (*n* = 37) were used for the evaluation of model performance in the Wales region only.

##### Environmental data

Environmental data were obtained for variables relating to land cover (UK Centre for Ecology and Hydrology's Land Cover Map 2015; www.ceh.ac.uk/services/land‐cover‐map‐2015, 25 m resolution), roads (Ordnance Survey (OS) Meridian™ 2; www.ordnancesurvey.co.uk, 10 m resolution), terrain (OS Terrain 50, 50 m resolution) and climate (Worldclim version 1; http://www.worldclim.org/, 1 km resolution). Environmental data were resampled to 100 m cell rasters to predict habitat suitability at a fine resolution, which was necessary for delineating irregularly shaped individual‐sized patches in the subsequent hybrid model (Appendix S1). The final model included six variables that were selected from a candidate list of 33 variables through a stepwise process of a priori selection, collinearity analysis and complexity optimisation (Appendix S1). These comprised three distance metrics: distance to nearest woodland (woodland distance), non‐woodland forage (forage distance) and urban areas (urban distance) as well as two variables based on the proportion of land cover within a 500 m radius (woodland cover and forage cover) and a categorical variable for land cover type (land cover, Appendix S1). Roe deer are known to occasionally occupy small green spaces in predominantly urban areas (Ciach & Fröhlich, 2019). Therefore, we used both categorical and proportional variables to include land cover information at the location where the species was recorded as well as the proportion of land cover within the local vicinity.

##### MaxEnt model parameterisation and validation

A fishnet grid of 10 × 10 km cells was created for each region. Background points for the development and validation of the MaxEnt model were only created within cells that intersected presence locations (England and Scotland; *n* = 908, Wales; *n* = 32, Appendix S1). The MaxEnt default of 10,000 background points was used for Wales (3.4 points/km^2^) and 100,000 points were used for England and Scotland (1.2 points/km^2^). Linear, quadratic, hinge and product feature classes were used as well as the default value of 1.0 for the regularisation multiplier (Appendix S1).

An *n*−1 cross‐validation technique was used to validate the MaxEnt model and to compare predictive performance between the populated and novel regions. The *n*−1 method trains a model on all data points (England and Scotland: *n* = 3843; Wales: *n* = 37) but one, then evaluates the model on that point and repeats until all points have been evaluated (Cawley & Talbot, 2003; Hijmans, 2012). Model performance was estimated based on the ability to correctly rank presences in the test data set higher than background points, as given by the mean area under the receiver‐operating‐characteristic curve (AUC). The AUC is a standard measure of goodness of fit that yields a value between 0.5 and 1, where 0.5 suggests the model performs no better than random and 1 indicates perfect prediction (Pearce & Ferrier, 2000). Values above 0.7 are generally considered an indication of good model fit (Hijmans, 2012). The use of the AUC metric to evaluate the performance of correlative models has been criticised (Jiménez‐Valverde, 2012; Lobo et al., 2008). However, we feel that its use in this study was appropriate as it facilitated a direct comparison of performance with previous studies (Acevedo et al., 2010; Croft et al., 2017, 2019) that were carried out for the same species and over the same spatial extent. Variable importance was assessed using a jackknife test, which measured the increase in regularised training gain when each variable was used in isolation and the decrease in gain when the variable was excluded from the full model (Phillips & Dudík, 2008). The relative contribution of each variable to the model was also estimated based on permutation importance, which is one of the metrics reported in the MaxEnt model output (Hijmans et al., 2017; Phillips & Dudík, 2008).

#### 2.2.2 Step 2. Generalising the habitat suitability map

**FIGURE 2:**
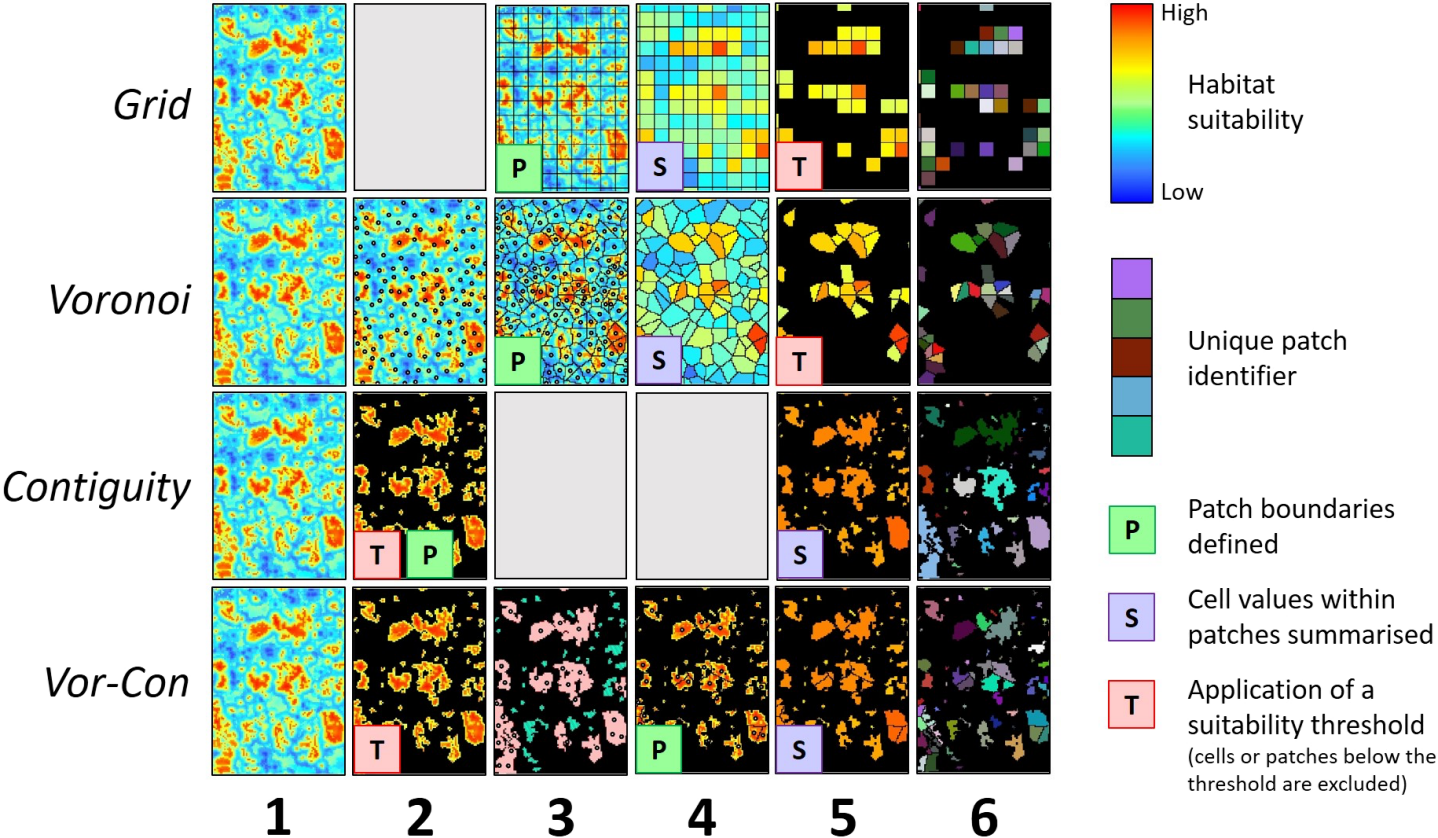
Stages of habitat suitability map (HSM) generalisation. Rows represent four generalisation methods used: Grid, Voronoi, Contiguity and Voronoi‐Contiguity (Vor‐Con). Columns denote stages in the generalisation process (see text for details). Common to all methods are stages (1) the original HSM, (5) the mean suitability of patches and (6) unique identifiers assigned to each patch. Key characteristics of the generalised map include (P) the definition of patch boundaries, (S) the summarisation of cell values within patches (i.e. calculating mean suitability) and (T) application of a suitability threshold to convert the HSM from continuous (i.e. low to high) to binary (i.e. suitable/not suitable) values. These characteristics may be defined at different developmental stages depending on the generalisation method used. Grey panels indicate the absence of a stage and are included for a more intuitive comparison of results at similar developmental stages across the four methods. Row Vor‐Con, column 3: red patches were divided using Voronoi polygons and green patches were unmodified.

There is currently no consensus on the most effective method of delineating patches from a habitat suitability map (HSM). We therefore evaluated four methods: (1) Grid, (2) Voronoi, (3) Contiguity and (4) Voronoi‐Contiguity (Vor‐Con) within the Historic Area of Expansion (HAE, Figure 1). The same key steps were used in each method: definition of patch boundaries (P), summarisation of the cell values within patches (S) and the application of a suitability threshold to eliminate patches or cells considered unsuitable (T, Figure 2). Applying a suitability threshold is required to convert cells of the HSM from continuous (i.e. low to high suitability) to binary (i.e. suitable/not suitable) values for patch delineation. A value of 0.56 was chosen as it maximised the sum of sensitivity and specificity in the MaxEnt model (Liu et al., 2005, 2016). The home range area of roe deer was assumed to be 0.06–1.5 km^2^ with an approximate average of 1 km^2^ (Coulon et al., 2008; Le Corre et al., 2008; Martin et al., 2018). Roe deer are generally solitary, males are territorial and both sexes demonstrate high home‐range fidelity (José & Lovari, 2010; Linnell & Andersen, 1998; Lovari et al., 2017). We therefore chose to delineate patches based on the home range area because it is biologically meaningful and appropriate for identifying relevant landscape features for management (e.g. individual woodlands).

##### Grid

The Grid method effectively resampled the HSM at a coarser resolution. A fishnet grid of 1 km^2^ cells was created for the extent of the HSM and the mean value of HSM cells within grid cells was calculated. Grid cells with a mean suitability below the threshold were removed (Figure 2, row Grid).

##### Voronoi

The Voronoi method used polygons to define patch boundaries, which were irregular polygons based on Voronoi tessellations (Holland et al., 2007). Point features were distributed across the extent of the HSM at an approximate density of 1 point/km^2^ (*n* = 60,350). Studies have shown that roe deer home ranges generally decrease with increasing population density and habitat quality (Kjellander et al., 2004; Saïd et al., 2009). To reflect this, points were distributed according to the probability distribution described by the HSM, which biased their location towards more suitable habitat (‘Create Spatially Balanced Points’ tool in ArcGIS, ESRI ArcMap Version 10.4.1). Therefore, point‐density increased and patch‐size decreased in relation to habitat suitability. A minimum distance of 150 m between points was used, which equalled the approximate radius of the lower limit of the home‐range area (0.06 km^2^). Voronoi polygons were created to define the geometry of patches (‘Create Thiessen Polygons’ tool in ArcGIS, ESRI ArcMap Version 10.4.1). Polygons were converted from a vector to a raster and then back to a vector to ensure patch boundaries aligned with cells of the HSM. The mean value of cells within patches was calculated and patches with a mean suitability below the threshold were removed (Figure 2, row Voronoi).

##### Contiguity

HSM cells with suitability values below the threshold were removed. Suitable cells that neighboured other suitable cells in any of the eight cardinal directions were considered part of the same patch (Figure 2, panel c). Patches smaller than the lower limit of the home‐range area (0.06 km^2^) were removed and the mean value of cells within the remaining patches was calculated (Figure 2, row Contiguity). This method produced patches that were larger than the upper limit of the home‐range area (1.5 km^2^). It was presented to demonstrate the importance of patch size in the case study and to illustrate the conceptual basis of the Vor‐Con method.

##### Voronoi‐Contiguity (Vor‐Con)

The Vor‐Con method included stages of both the Voronoi and Contiguity methods. Patches were created using the Contiguity method and grouped into the following classes based on the observed limits of the home‐range area: small (<0.06 km^2^), medium (0.06–1 km^2^) and large (>1 km^2^). Small patches were removed and medium patches were not modified. Large patches were divided into smaller patches using Voronoi polygons following the same procedure as the Voronoi method. Point features were created at an approximate density of 1 point per km^2^ (*n* = 11,146). The points were distributed according to the probability distribution described by the HSM, using only the cells within the boundaries of large patches. Voronoi polygons were created and converted from a vector to a raster and then back to a vector to ensure patch boundaries aligned with cells of the HSM. The mean value of cells within patches was calculated (Figure 2, row Vor‐Con).

Suitability values in the generalised maps (Figure 2, column 5) were scaled by multiplying by 100 and rounding to integers as a formatting requirement of the RangeShifter software. Patches were also assigned a unique identification number (Figure 2, column 6).

#### 2.2.3 Step 3. Simulating patch occupancy

##### Parameterising the hybrid model

The RangeShifter platform was designed to use extensive demographic information (survival rates, fecundity, maximum age, etc.) to simulate range expansions as a function of stochastic interactions between individuals and the environment (Bocedi, Palmer, et al., 2014). However, in this study, we simplified the modelling of population dynamics in RangeShifter to reduce data requirements. Demographic parameters were standardised and constrained by density dependence acting at the patch level so that emigration and immigration rates were dependent on patch size and quality. Regional‐scale patterns of population spread therefore emerged solely as a function of the size, quality and connectivity of individual‐sized patches.

##### Simplifying population dynamics

The hybrid model was structured as follows: (i) occupied patches produced a number of dispersers proportional to patch size and quality, (ii) dispersers interacted with the landscape to transfer between patches and (iii) dispersers settled in patches occupied below carrying capacity. This was implemented in RangeShifter as an asexual stage‐structured population model based on a Leslie transition matrix (Bocedi, Palmer, et al., 2014). Three stage classes were considered: juveniles (<1 year old), dispersers (1 year old) and adults (≥2 years old). All surviving individuals develop to the next stage class and only adults are able to reproduce. The following transition matrix (derived from Bocedi, Palmer, et al., 2014) was applied:

$$\left[\begin{array}{ccc} 0 & 0 & Ф\\ \mathrm{Sj} & 0 & 0\\ 0 & \mathrm{Sy} & \mathrm{Sa} \end{array}\right],$$

where fecundity Ф and the survival probabilities of juveniles Sj, disperses Sy and adults Sa were set to a standardised value of 1. The maximum age of adults was set to 1000, so that occupied patches were likely to remain occupied throughout the simulation (i.e. the probability of local population extinction was close to 0). Density dependence acted on survival and fecundity and was implemented in RangeShifter as an exponential decay:

(1) $$x_{i}=x_{i,0}*e^{-{\mathrm{bN}}_{t}},$$

where $x_{i}$ is a parameter for survival or fecundity, $x_{i,0}$ is the maximum value of the parameter at low densities, *b* is the strength of density dependence and $N_{t}$ is the total number of individuals in the local population at time *t* (derived from Bocedi, Palmer, et al., 2014, RangeShifter user manual). The strength of density dependence coefficient, 1/*b*, was also set to a standardised value of 1.0. Habitat suitability was assumed to be constant during the simulation period and linearly related to carrying capacity. Using standardised parameter values (Table 1) and incorporating density dependence in the hybrid model established a relatively simple set of rules for determining patch occupancy. The model assumes that over time more dispersers are likely to emerge from, and settle in, larger, more suitable patches than smaller, less suitable patches.

**TABLE 1:** Summary of parameters used in the RangeShifter (Bocedi, Palmer, et al., 2014) model.

	Model parameter	Symbol	Estimate
Population dynamics	Strength of density dependence coefficient (1/*b*)		1.0
	Stage classes (minimum age)		Juveniles (0) Dispersers (1) Adults (2)
	Maximum age (years)		1000
	Probability of reproduction		1.0
	Fecundity^a^	Ф	1.0
	Survival rates^a^		
	Juveniles	Sj	1.0
	Dispersers	Sy	1.0
	Adults	Sa	1.0
Dispersal	Emigration probability	Dy	1.0
	Movement parameters		
	Perceptual range	PR	400 m
	Directional persistence	DP	5
	Settlement probability		
	Slope (*α*)	Ps	−100
	Inflexion point (*β*)		1.0
	Maximum number of steps (Euclidean distance)		200

^a^ Parameters constrained by density dependence.

##### Dispersal

Dispersal between patches was modelled as three discrete phases of emigration, transfer and settlement. All juveniles that survived and developed into dispersers, emigrated from their natal patch. Movement during the transfer phase was modelled at the finer scale (0.01 km^2^) of the scaled HSM using the embedded Stochastic Movement Simulator (SMS). The SMS simulated movement as a series of discrete nearest‐neighbour steps across a cost surface, similar to the Least Cost Path (Bocedi, Palmer, et al., 2014; Palmer et al., 2011). The cost surface was derived by inverting the scaled HSM using the formula: *100 – values of the scaled HSM* (‘Raster calculator’ tool in ArcGIS, ESRI ArcMap Version 10.4.1), which assumed movement costs were inversely related to habitat suitability. Dispersers would therefore be less likely to move through low‐quality habitat.

The transfer phase was influenced by parameters describing the maximum number of steps, the perceptual range (PR) of the species and their tendency to follow a correlated random walk, defined as directional persistence (DP, Table 1). The perceptual range was estimated to be 400 m from habitat selection studies based on global positioning system (GPS) telemetry data (Coulon et al., 2008). A value of five was used for directional persistence simulating a moderate tendency for the animal to follow correlated paths within the landscape. Dispersers could move a maximum of 200 steps which equates to a Euclidean distance of 20 km (Debeffe et al., 2013; Wahlström & Liberg, 1995).

##### Estimating patch occupancy

**FIGURE 3:**
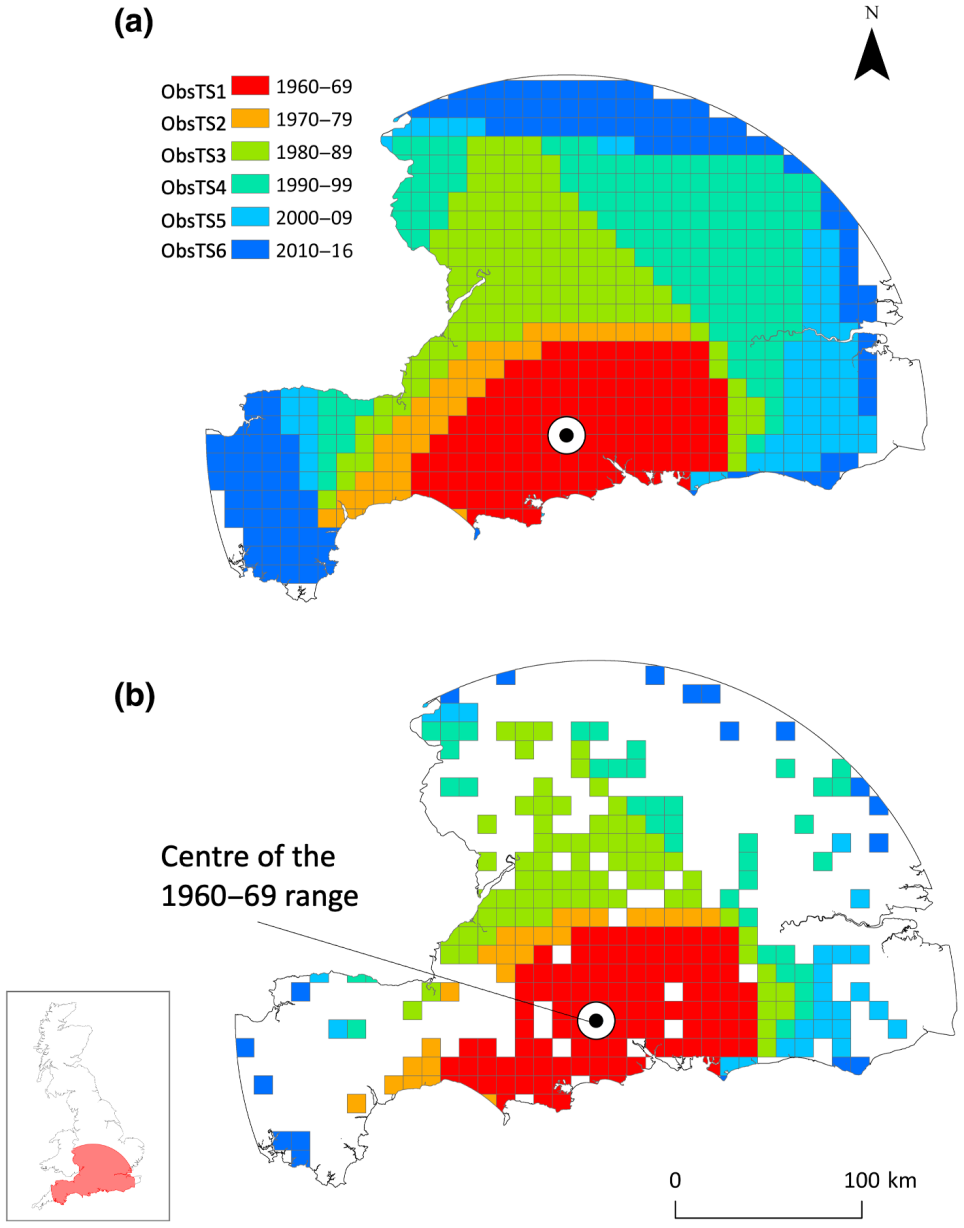
Patterns of observed roe deer (*Capreolus capreolus*) range expansion in the historic area of expansion (HAE) across six timesteps (ObsTS) from 1960 to 2016 used to evaluate the hybrid models, described as (a) ObsRange: the observed range estimated from minimum convex polygons created around presences and (b) ObsPresences: presence locations within the observed range. Inset: location of the HAE in Great Britain.

Distribution data from 1960 to 2016 within the HAE (Figure 3) were divided into five 10‐year periods (1960–2009) and one 7‐year period (2010–2016), described as Observed Timesteps (ObsTS1‐ObsTS6). Simulations were initialized with the species occupying patches within a 10 km radius buffer around the centre of the observed range at ObsTS1 (Figure 3; see Appendix S2 for initialization parameters). A total of 10 simulations were run for a sufficient time to achieve complete occupation of all available patches, which was estimated from preliminary trials. In each simulation, patch occupancy (1 = occupied, 0 = not occupied) was estimated at six regular time intervals, defined as Simulated Timesteps (SimTS1‐SimTS6). Mean patch occupancy at each SimTS was calculated as the mean occupancy from the 10 simulations. A threshold value for mean patch occupancy of 0.7 was applied (i.e. patches predicted to be occupied in 7 out of 10 simulations were considered occupied). Application of a threshold was necessary to convert mean patch occupancy from continuous (i.e. 0 to 1) to binary (i.e. 0 = not occupied, 1 = occupied) values. Cells of the 10 × 10 km grid that intersected occupied patches defined the simulated species range at each SimTS.

#### 2.2.4 Step 4. Model evaluation

Performance of the hybrid models was assessed based on the ability to recreate observed patterns of historic population spread. A 10 × 10 km grid of regular cells was created for the HAE, which defined the regional scale of model evaluation. Minimum convex polygons (Meyer et al., 2017) were constructed around presences at each timestep in ArcGIS (ESRI ArcMap Version 10.4.1). Grid cells that intersected polygons were used to define the observed species range (ObsRange, Figure 3a). Although convenient, this method is prone to overestimating the species range by including areas of unsuitable habitat (Burgman & Fox, 2003). We therefore also identified presence locations within the observed range for a more comprehensive evaluation of model performance. Grid cells within the species range at each timestep that intersected presences were defined as ‘ObsPresences’ (Figure 3b).

Model performance was assessed by comparing the simulated species range to ObsRange and ObsPresences at matching timesteps (e.g. SimTS1/ObsTS1) and calculating the True Skill Statistic (TSS, Allouche et al., 2006), which is the sum of model sensitivity (the proportion of predicted presences that were correct), and specificity (the proportion of predicted absences that were correct), minus one. The TSS ranges from −1 to 1, and good predictive performance is indicated by values >0.4 (Allouche et al., 2006; Eskildsen et al., 2013; Landis & Koch, 1977). Overall model performance was based on the mean of the TSS values from the six timesteps for ObsRange and ObsPresences. Finally, a sensitivity analysis was performed to assess the impact of the three user‐defined parameters (perceptual range, directional persistence and maximum number of steps, varied by ±10%) on the simulated species ranges.

#### 2.2.5 Step 5. Applying the model to a novel region

Estimates of habitat suitability in Wales were projected from the England and Scotland region using the same set of environmental variables. All values for environmental variables in the Wales region were within the limits of the England and Scotland regions. The projected HSM for Wales was generalised into a landscape of individual‐sized patches using the Vor‐Con method. It was also inverted to be used as a cost surface in the hybrid model using the formula described above (see ‘Dispersal’ section of Step 3). A 10 × 10 km grid was created for Wales. The hybrid model was parameterised with the same parameter set used for the populated region and initialised with the species occupying patches within grid cells that intersected observations of species presence (Appendix S2). Patch occupancy was estimated at 10 simulated timesteps (SimTS1‐SimTS10).

## 3 RESULTS

### 3.1 Habitat suitability

**FIGURE 4:**
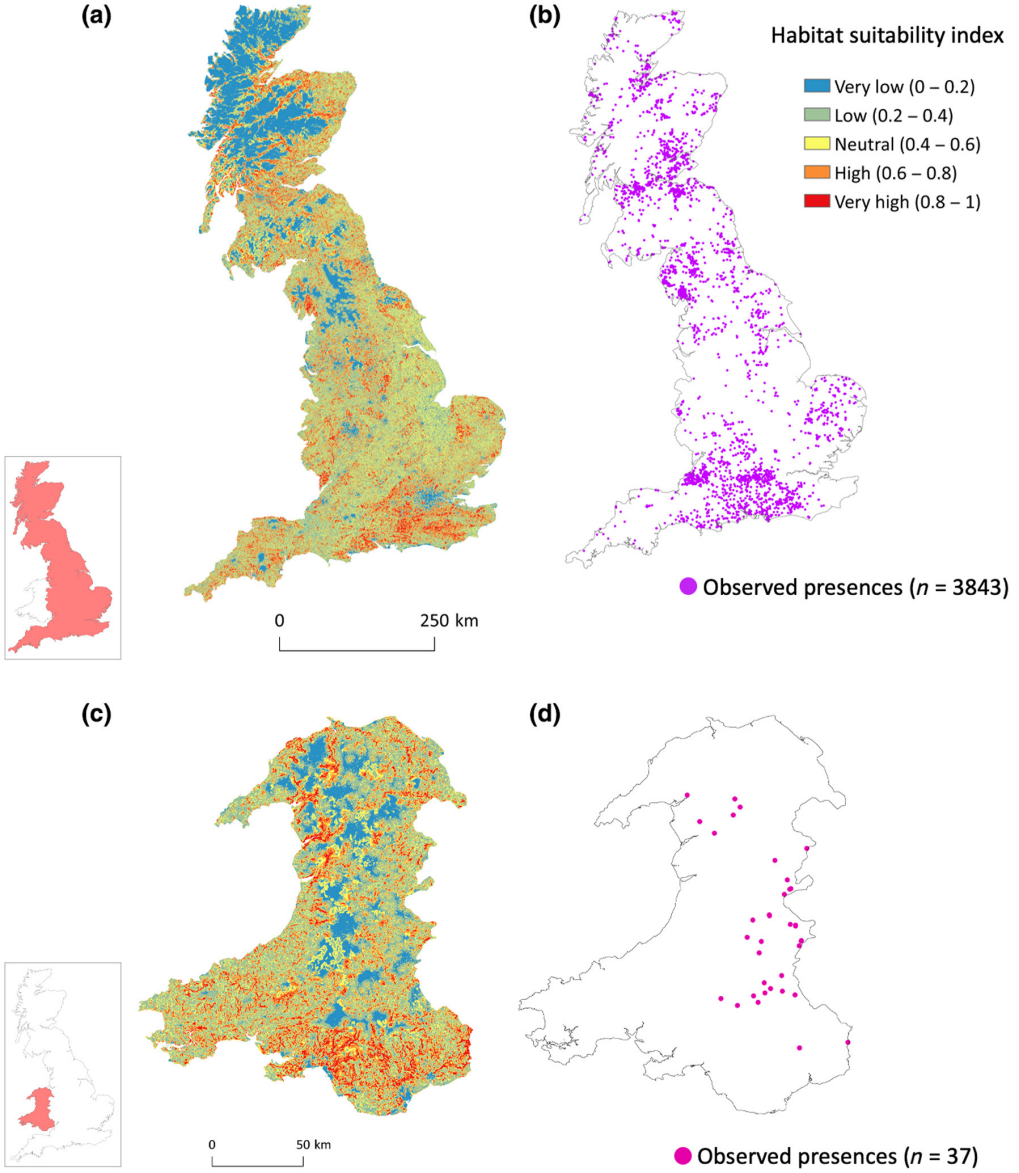
Predicted suitability of habitat for roe deer (*Capreolus capreolus*) from the MaxEnt model in (a) England and Scotland and (c) Wales. Maps (b) and (d) show the locations of observed presences in England and Scotland (*n* = 3843) and Wales (*n* = 37), respectively (for data sources, see Appendix S1). Inset maps show the locations of each region in Great Britain.

**FIGURE 5:**
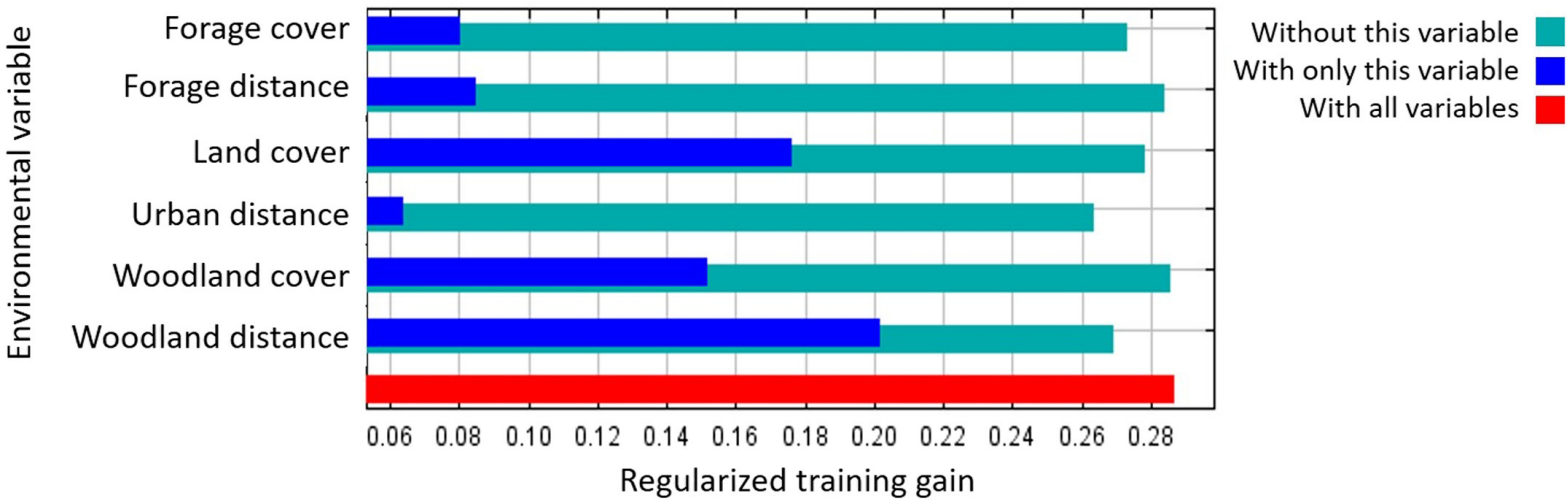
Importance of environmental variables to the predictions of habitat suitability derived from the best‐performing MaxEnt model for England and Scotland assessed using a jackknife test (Elith et al., 2006).

Figure 4 shows the predicted suitability of habitat for roe deer in England and Scotland (Figure 4a) and Wales (Figure 4c). The area under the receiver‐operating characteristic curve (AUC) values from *n*−1 cross‐validation indicated that the correlative MaxEnt model performed well (AUC > 0.7) in both England and Scotland (0.72 ± 0.26, mean ± standard deviation) and Wales (0.76 ± 0.25) regions. Three variables: woodland distance, land cover and woodland cover achieved the highest regularised training gain when isolated in the jackknife test (Figure 5) and had a combined relative contribution of 84.5% to the full model (Table 2). Forage distance, forage cover and urban distance provided minimal gain (Figure 5) and collectively contributed 15.5% (Table 2). Results from omitting each variable showed that urban distance and woodland distance contained the most information not contained in the other variables (Figure 5).

**TABLE 2:** Relative contribution based on permutation importance of environmental variables in the best‐performing MaxEnt habitat suitability model for roe deer (*Capreolus capreolus*) in England and Scotland.

Environmental variable	Relative contribution (%)
Woodland distance	73.8
Urban distance	11.8
Land cover	8.5
Forage cover	3.7
Woodland cover	2.2
Forage distance	0.1

### 3.2 Model performance in the historic area of expansion

**FIGURE 6:**
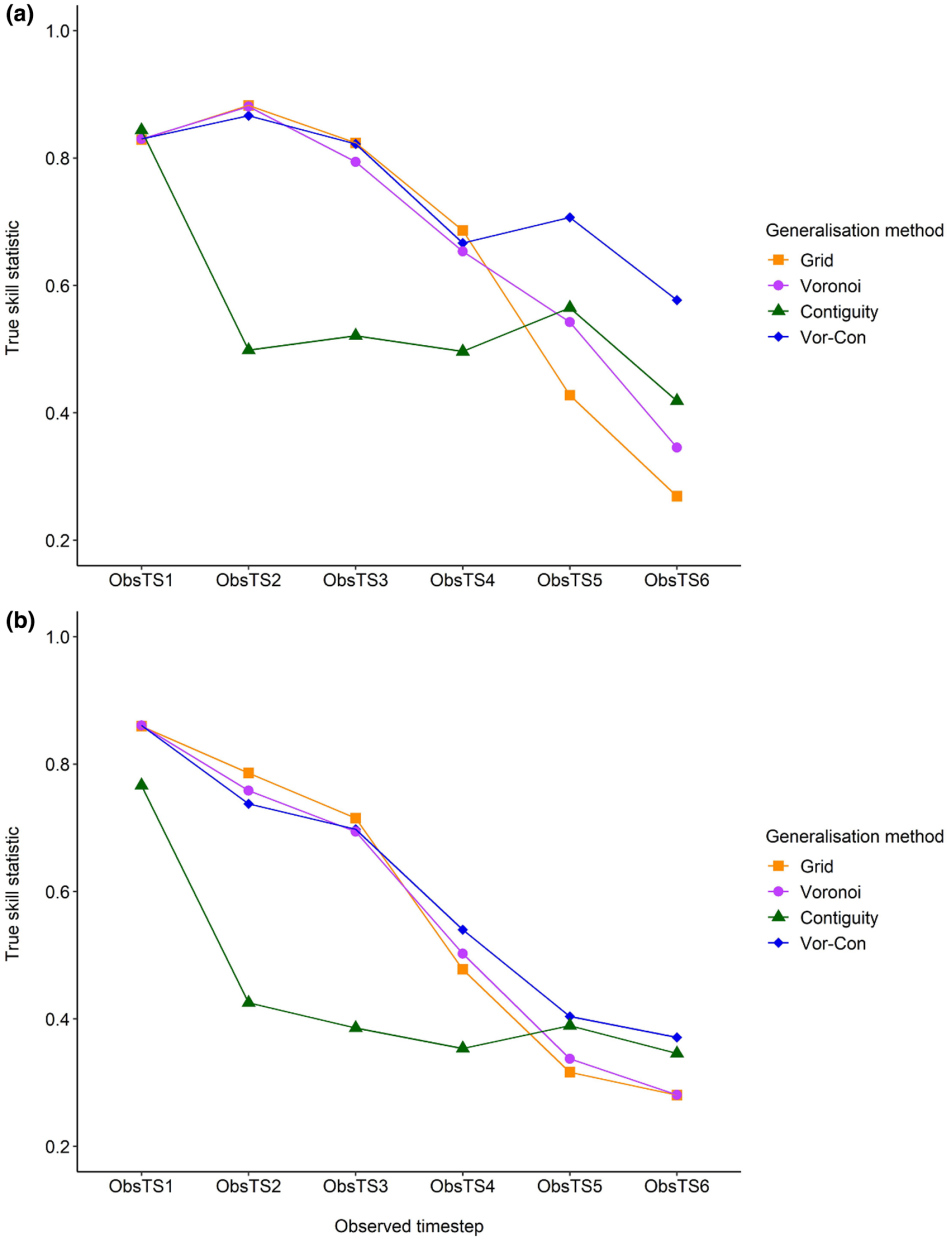
Results for the hybrid models showing the spatial agreement (True Skill Statistic, Allouche et al., 2006) between the simulated range and (a) the observed species range (ObsRange) and (b) the distribution of presences within the observed range (ObsPresences) at six timesteps (ObsTS) for roe deer (*Capreolus capreolus*) in the Historic Area of Expansion (HAE) from 1960 to 2016.

**FIGURE 7:**
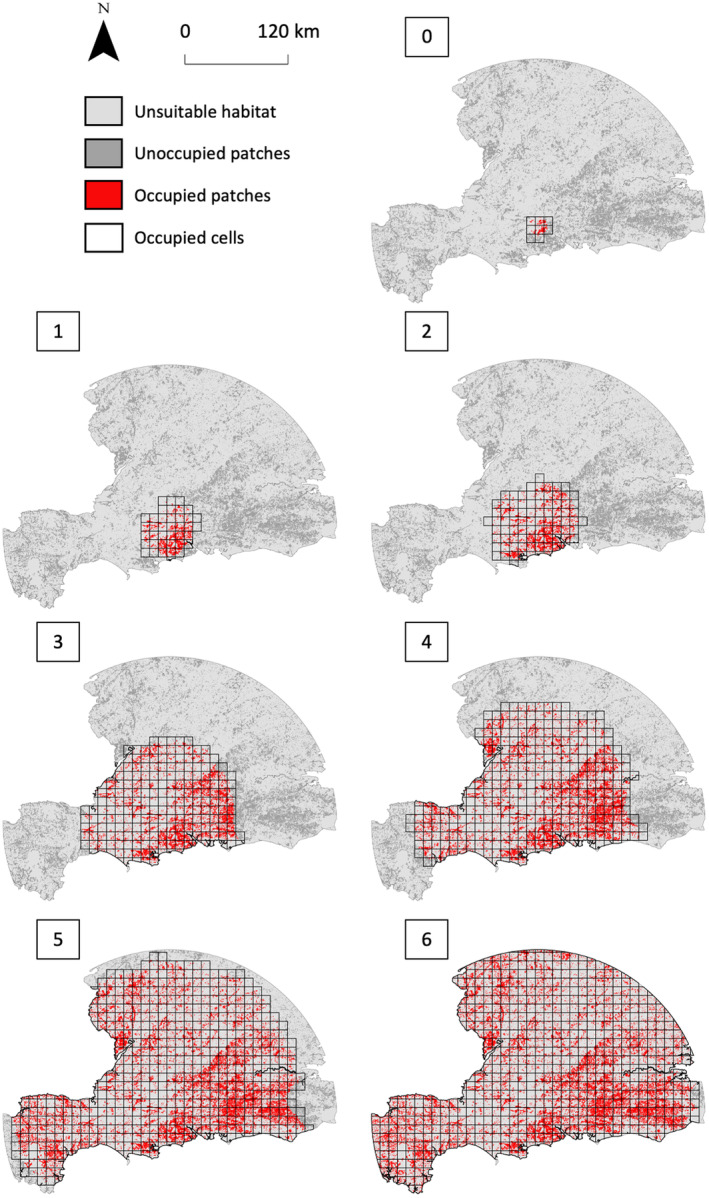
Predicted patterns of roe deer (*Capreolus capreolus*) range expansion in the historic area of expansion (HAE) from the highest‐performing (Vor‐Con) hybrid model. Numbers indicate simulated timesteps from initialisation (0) to near‐total occupation of suitable patches (6). The 10 × 10 cells (occupied cells) were compared to ObsRange and ObsPresences for model evaluation (see Section 2 for details).

All the hybrid models performed well in recreating patterns of population spread from the historic area of expansion (HAE), as indicated by mean TSS values >0.4 when simulations were compared to the observed species range (ObsRange) and to the distribution of species presences (ObsPresences, Table 3). The observed patterns of population spread in the HAE were most accurately predicted using the Vor‐Con method (Table 3). The Grid and Voronoi methods also achieved good spatial agreement between observed and simulated ranges (Table 3). The Contiguity method was the least accurate with the lowest TSS values in all of the analyses (Table 3). A visual inspection of model outputs indicated that the Contiguity method overestimated the species range and showed lower‐than‐average sensitivity values (a higher proportion of false presences, Appendix S2). The performance of all models decreased over time (Figure 6), which was an expected result of the method used for model evaluation (see Section 4 and Appendix S2 for more information). Predictions of the species range using the highest‐performing (Vor‐Con) method (Figure 7) were insensitive to variation in any of the three user‐defined parameters (perceptual range, directional persistence and maximum number of steps, varied by ±10%; Appendix S2).

**TABLE 3:** Evaluation results for the hybrid models showing the spatial agreement (True Skill Statistic, Allouche et al., 2006) between the simulated range and (i) the observed species range (ObsRange) and (ii) the distribution of presences within the observed range (ObsPresences) at six timesteps (ObsTS) for roe deer (*Capreolus capreolus*) in the historic area of expansion (HAE) from 1960 to 2016.

Comparator	Generalisation method	True skill statistic			
		Mean	SD	Min.	Max.
ObsRange	Grid	0.65	0.25	0.27	0.88
	Voronoi	0.67	0.20	0.35	0.88
	Contiguity	0.56	0.15	0.42	0.84
	**Vor‐Con**	**0.74**	**0.11**	**0.58**	**0.87**
ObsPresences	Grid	0.57	0.25	0.28	0.86
	Voronoi	0.57	0.24	0.28	0.86
	Contiguity	0.44	0.16	0.35	0.77
	**Vor‐Con**	**0.60**	**0.20**	**0.37**	**0.86**

*Note*: Bold text indicates the highest‐performing model. Values given are the mean, standard deviation (SD), minimum (Min.) and maximum (Max.) TSS scores across the six timesteps.

### 3.3 Range expansion in Wales

**FIGURE 8:**
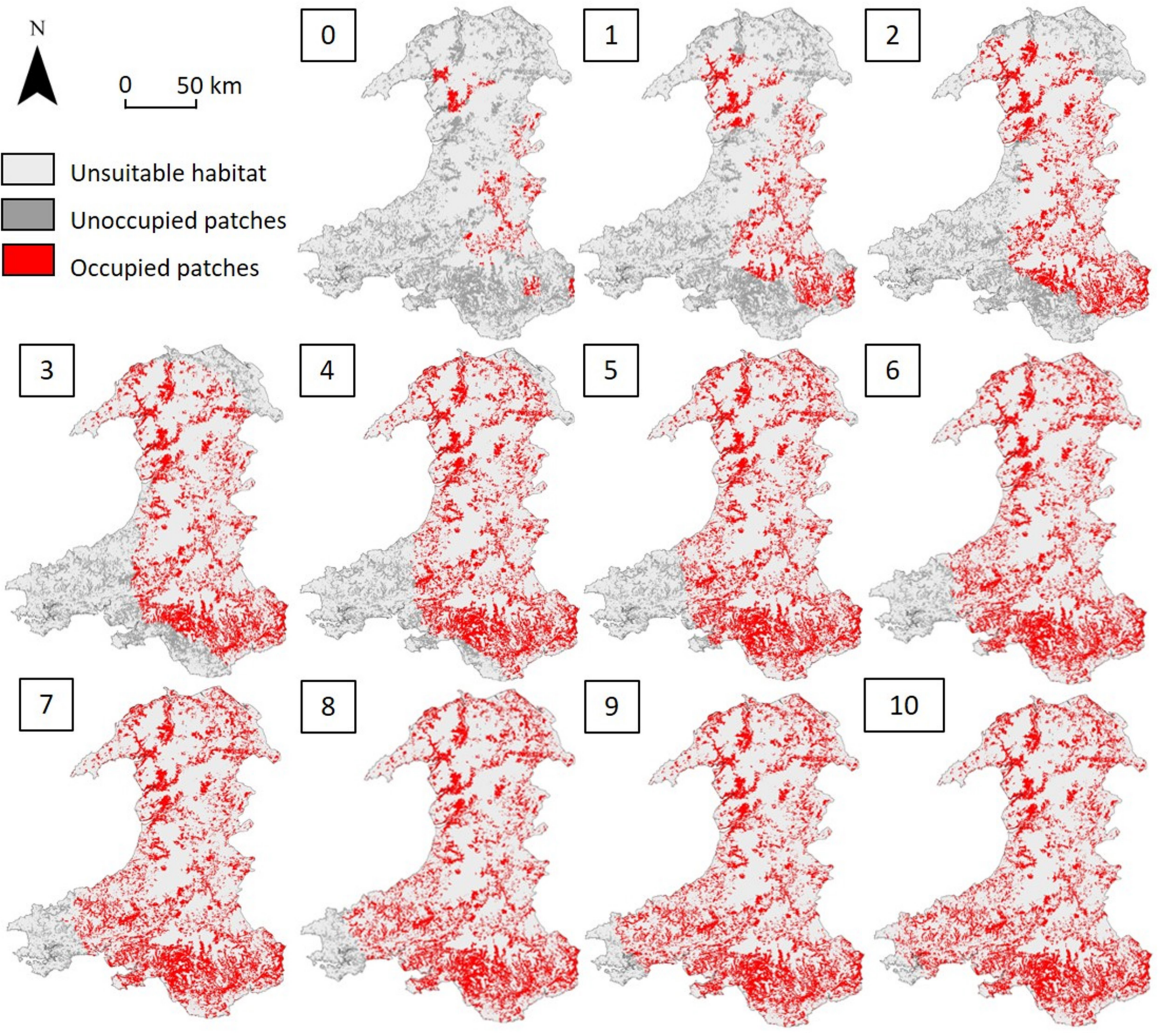
Predicted patterns of roe deer (*Capreolus capreolus*) range expansion in Wales from the (Vor‐Con) hybrid model. Numbers indicate simulated timesteps from initialisation (0) to near‐total occupation of suitable patches (10). The initial distribution of occupied patches was based on observed presences.

Suitable habitat patches (suitability ≥0.56) covered approximately 26% (5268 km^2^) of the total area. The population is predicted to spread through the centre of Wales, initially progressing from east to west. The range front is estimated to advance in the northern half of Wales towards the northeast and in the southern half of the country towards the southwest (Figure 8, 1–4). Once the population reaches the southern coastline, expansion is predicted to gradually continue west (Figure 8, 5–10).

## 4 DISCUSSION

We developed a hybrid species distribution model to predict regional‐scale patterns of population spread for an animal species from limited distribution and demographic data. A correlative, MaxEnt model (Phillips et al., 2017) was constructed using presence‐only occurrence data for roe deer in mainland Great Britain. The model estimated the suitability of habitat from a populated region (England and Scotland) and predicted the potential future range of the population in a novel region (Wales). The habitat suitability map was then generalised into a landscape of individual‐sized patches using a range of methods and used to represent the environment in a hybrid model to make dynamic predictions of population spread. The hybrid model was evaluated against historical species distribution changes and applied to predict the spatial patterns and relative timing of colonisation events of roe deer in Wales.

### 4.1 Habitat suitability

The results from the *n*−1 cross‐validation showed that the MaxEnt model performed well in both the populated and novel regions. The area under the receiver operating curve (AUC) values attained in this study are similar to those reported from previous studies of roe deer in the UK by Acevedo et al. (2010) (0.85), Croft et al. (2017) (0.64) and Croft et al. (2019) (0.9). Furthermore, our correlative model was validated to a spatial resolution (0.01 km^2^) that is a 100 times finer than that used by Croft et al. (2017) (1 km^2^) and 10,000 times finer than Acevedo et al. (2010) and Croft et al. (2019) (100 km^2^). The high resolution of the output was critical to subsequent modelling stages, as it allowed the suitability map to be generalised into a landscape of patches based on the home range area of an individual roe deer. This captured structural details of the landscape, such as the size, distribution and geometry of habitat patches, that are important in shaping patterns of population spread (Wilson et al., 2010; Wilson, Davies, et al., 2009; Wilson, Dormontt, et al., 2009). Estimating habitat suitability also provided insights into the species‐environment relationship that was essential in characterising the environment for the roe deer, a generalist species, whose distribution is known to be influenced by a range of habitat types (Croft et al., 2019; Jepsen & Topping, 2004; Kilheffer & Underwood, 2018).

### 4.2 Hybrid model performance

We evaluated the hybrid models and tested four methods of generalising the habitat suitability map from the MaxEnt model using historic distribution data. The approach is similar to Singer et al. (2018) but uses presence‐only rather than presence/absence data, which was simple to implement and relatively straightforward to interpret. The Grid and Voronoi methods performed well and achieved a high level of spatial agreement between simulated and observed ranges. In both methods, patch geometry was pre‐defined, as in traditional grid‐based models (Bian, 2003; McLane et al., 2011). For the Contiguity and Vor‐Con methods, a suitability threshold was applied to the HSM as the first step, which retained the natural geometry of landscape features (Girvetz & Greco, 2007). The model based on the Vor‐Con method achieved the best performance of the four methods, whereas the model based on the Contiguity method showed the worst performance. As the only technique that did not constrain patches to the size of an individual home range, the poor performance of the Contiguity method is likely due to a phenomenon known as the ‘mega patch problem’ (Cavanaugh et al., 2014). This issue arises because, when a patch is occupied, individuals within that patch are effectively omnipresent and so instantaneously traverse the length of the patch, which can result in an overestimation of spread through larger patches. This issue was resolved in the Vor‐Con method by the division of large patches using Voronoi polygons. Although a variety of patch‐delineation models are available (e.g. Cavanaugh et al., 2014; Girvetz & Greco, 2007; Kilheffer & Underwood, 2018), the methods used in this study were selected to minimise model complexity and improve the accessibility of the model to wildlife management practitioners (Addison et al., 2013; Guisan et al., 2013; Tulloch, Sutcliffe, et al., 2016).

### 4.3 Simplifying population dynamics

Our modelling approach reduced the demand for demographic data usually associated with parameterising a hybrid model by simplifying the simulation of population dynamics. Using generic rules to describe population dynamics is conceptually similar to a stochastic patch occupancy or traditional grid‐based model (Bian, 2003; Hanski & Ovaskainen, 2003; Preisler et al., 2004). The key strength of our approach is in the more sophisticated modelling of dispersal, which was facilitated by the RangeShifter platform (Bocedi, Palmer, et al., 2014). The embedded Stochastic Movement Simulator (SMS) in RangeShifter enabled us to predict regional‐scale patterns of population spread as a function of patch characteristics (i.e. the size, geometry and composition of patches) and landscape structure (i.e. the distribution and connectivity of patches [Bocedi, Palmer, et al., 2014; Palmer et al., 2011]). The SMS provides an important advantage in modelling the range expansion of animal species, such as roe deer, with dispersal expected to be influenced by key properties of the landscape (e.g. land cover, elevation etc., Debeffe et al., 2013; Wahlström & Liberg, 1995). Our approach could easily be applied to predict distribution changes for a wide range of taxa using the same set of parameters for population dynamics (i.e. standardised values) and species‐specific parameters for dispersal (perceptual range, directional persistence and maximum number of steps, see Methods section for details).

### 4.4 Model assumptions

When interpreting the results from our study and considering our approach for future applications, it is important to understand the implications of two key assumptions that were made. Firstly, to simplify the modelling of population dynamics, it was necessary to assume that the roe deer population would inevitably spread and realise its potential range (i.e. expansion was certain). We feel that this was reasonable based on historic patterns of expansion in Great Britain and Europe and the biological characteristics of the species. The environmental conditions in Wales are similar to England and Scotland, where the roe deer population is widely distributed. The rates of annual survival and reproduction are also high for roe deer, so local extinctions are unlikely (Cobben et al., 2009; Davis et al., 2016; Flajšman et al., 2013; Gaillard et al., 1993; Wäber et al., 2013). However, it should be noted that a wide range of additional factors may influence the likelihood of their expansion in Wales, such as human activity, interspecific interactions and climate change (Dormann et al., 2012; Pacifici et al., 2020).

Secondly, it was assumed that habitat suitability was the only factor driving patterns of population spread. Predictions of population spread from the hybrid model were estimated based on the limited set of environmental variables used to construct the underlying habitat suitability map. Because the modelled environment was static, it failed to account for temporal variation in variables, such as land use and climate. Historic temporal variation may reduce the accuracy of the habitat suitability map as environmental conditions at the point of species presence may have changed since the time of recording. Predictions of future population spread also assume that the environment will remain in its current state, which may be inaccurate. Developing methods to incorporate dynamic environments in models of population spread is a subject of ongoing research (Lecocq et al., 2019; Milanesi et al., 2020) and is a priority for future adaptation of the model.

Validating and assessing the performance of simulation‐based models also presents a methodological challenge (Zurell et al., 2022). While our evaluation method quantified the relative performance of the hybrid models, comparing our results to an independent dataset would facilitate more robust model validation and assessment of absolute performance. Presence‐absence species distribution data would be particularly valuable, as they provide a similar level of information to the model output. In contrast, our evaluations were made using maps derived from presence‐only distribution data (ObsRange and ObsPresences). ObsRange was constructed from minimum convex polygons, which most likely overestimated the species range (Burgman & Fox, 2003). Conversely, ObsPresences represented a limited number of locations within the species range where observations were recorded. Therefore, model outputs were more likely to be penalised for under‐prediction (i.e. low specificity) and over‐prediction (i.e. low sensitivity) when compared to ObsRange and ObsPresences, respectively (Appendix S2). The magnitude of penalisation increases at each timestep, as the extent of the predicted range becomes larger relative to the total area, which results in a decrease in model performance over time (Appendix S2).

High‐quality independent presence/absence data are rarely available for validating dynamic models of population spread. However, technological advancements such as unmanned aerial vehicles provide novel opportunities to collect higher quality presence/absence distribution data across large spatial extents, which would facilitate more robust model validation (Anderson & Gaston, 2013; Baxter & Hamilton, 2018). Furthermore, higher‐quality data would also improve the accuracy of simulating movement during the transfer phase of dispersal. Describing movement using a cost surface derived from a habitat suitability map assumes movement is influenced by the same environmental variables that drive species distributions. In reality, variables such as elevation and annual rainfall may have an equal effect on distributions but are likely to offer different levels of resistance to movement. Future studies may look to incorporate radio tracking or global positioning system (GPS) telemetry data to derive more accurate cost surfaces from observations of movement behaviour (Diaz et al., 2021).

### 4.5 Roe deer in Wales

Roe deer are the most widespread deer species in Europe with Great Britain being one of many countries where numbers are increasing rapidly (Croft et al., 2019; Linnell et al., 2020; Ward, 2005). Restoring the population in Wales is an important conservation opportunity. However, it is essential that numbers are maintained at a level that does not place unsustainable pressure on the environment (Apollonio et al., 2010; Carpio et al., 2021). As roe deer have already started to spread from England into Wales, there is a pressing need to proactively develop regional‐ and local‐scale management strategies. Detection at an early stage of colonisation increases the likelihood of successfully controlling population sizes and decreases the long‐term costs of management (Aschim & Brook, 2019; Guisan et al., 2013). Physical monitoring techniques are usually geographically limited, due to the costs and logistics of fieldwork and specialist equipment. At the regional scale, our model can be used to prioritise areas for surveillance and guide early management actions, such as the engagement of landowners, construction of protective fencing and establishment of deer management groups. If sightings are recorded in a novel area, our model reveals where in the neighbouring region populations are most likely to spread to. The use of individual‐sized patches in our model further benefits local‐scale decision‐making by enabling the identification of specific parcels of the landscape for targeted management. As surveillance yields more data on the species' distribution, the model can be adapted to support long‐term population management.

## 5 CONCLUSIONS

Data limitations are a key challenge in developing predictive models that can support local and regional‐scale wildlife management strategies. Often, decision‐makers must allocate resources based on expert knowledge, coarse‐level estimates of species distributions and overly simplistic models of population spread. We present a relatively straightforward modelling approach that provides managers with a cost‐effective, evidence‐based tool for guiding actions and detecting expanding animal populations at an early stage of colonisation. Our approach fills an urgent need for a dynamic model that can be constructed with limited data, is accessible to wildlife managers and can be adapted to suit a wide range of taxa.

## AUTHOR CONTRIBUTIONS

**Owain Barton:** Conceptualization (equal); formal analysis (lead); investigation (lead); methodology (lead); writing – original draft (lead); writing – review and editing (equal). **John R. Healey:** Conceptualization (supporting); supervision (supporting); writing – review and editing (supporting). **Line S. Cordes:** Conceptualization (supporting); formal analysis (supporting); methodology (supporting); supervision (supporting); visualization (supporting); writing – review and editing (supporting). **Andrew J. Davies:** Formal analysis (supporting); methodology (supporting). **Graeme Shannon:** Conceptualization (equal); funding acquisition (lead); investigation (supporting); methodology (supporting); supervision (lead); writing – original draft (supporting); writing – review and editing (equal).

## CONFLICT OF INTEREST STATEMENT

The authors declare that no competing interests exist.

## Supporting information


Appendix S1

## Data Availability

Species distribution data used in this study are available by digital request from the National Biodiversity Network Atlas (www.nbnatlas.org, formerly NBN Gateway), the Biodiversity Information Service (www.bis.org.uk), the Wildlife Trust of South West Wales (www.welshwildlife.org) and the North Wales Environmental Information Service (www.cofnod.org.uk). Environmental data for variables relating to land cover, roads and terrain are accessible via Digimap (ww.digimap.edina.ac.uk). Climate data are available from WorldClim (www.worldclim.org).
