# Supplementary material for: Predicting the spatial expansion of an animal population with presence‐only data
Source: Ecol Evol. 2023 Nov 27;13(11):e10778. doi: 10.1002/ece3.10778 (PMC10681852; doi:10.1002/ece3.10778)
Supplement: Supplementary file 1 — Appendix S1 [file ECE3-13-e10778-s001.docx]

# Appendices

## S1. MaxEnt habitat suitability model

### Data preparation

### Species occurrences

(1) England and Scotland

- Roe deer occurrence data recorded to a precision of 100 m were downloaded for the UK mainland from the National Biodiversity Network Gateway (www.nbnatlas.org, formerly NBN Gateway)
- Years range: 1953 to 2016 (n = 5,653: no date (excluded) = 16; 1950-59 = 3; 1960-69 = 469; 1970-79 = 40; 1980-89 = 117; 1990-99 = 462; 2000-09 = 1026; 2010-16 = 3520)
- Removed duplicates, (n = 4,537)
- Of which were in England and Scotland, (n = 4,516)
- Removed points within 100 m of roads (n = 3,953). It was assumed that these probably originated from deer-vehicle collisions (DVCs) which may be reported more often than incidental sightings and could introduce sampling bias towards roads if included
- Removed points with incomplete environmental data for all variables (n = 3,843)
- Final number of presence points used in England and Scotland (n = 3,843)

(2) Wales

- Presences in Wales (n = 21) were augmented by data obtained from the Biodiversity Information Service (www.bis.org.uk, n = 124), the Wildlife Trust of South West Wales (www.welshwildlife.org, n = 4) and the North Wales Environmental Information Service (www.cofnod.org.uk, n = 32)
- Total presences in Wales (n = 181)
- Removed duplicates (n = 124)
- Selected points for the mainland of Wales only (n = 121)
- Removed points within 100 m of roads (n = 106)
- To avoid potentially overestimating the current range of roe deer in Wales, only records that included a description of a direct visual observation (i.e., a sighting or photograph) were used for model evaluation (n = 37). Records missing a description or indirect observations (i.e. pellets, footprints, barking etc.) were excluded (n = 69).
- Final number of presence points used in Wales (n = 37)

### Background sampling

(1) Extent

- The species data were collected from a wide range of sources and sampling effort was indeterminable
- It was assumed to be unlikely that roe deer are truly in equilibrium with their environment across all of England and Scotland (Croft et al., 2019; Elith & Leathwick, 2009; Ward, 2005)
- Background sampling was therefore spatially restricted to satisfy the basic assumptions of species distribution modelling and to account for spatial sampling bias in the species data (Elith & Leathwick, 2009; Fourcade et al., 2014; Phillips et al., 2009)
- A fishnet grid of 10 x 10 km cells was created for each region
- For both regions, background samples for all model development and evaluation were only taken within those 10 x 10 km cells that intersected presence data (England and Scotland; n = 908, Wales; n = 32)

(2) Number of background points

- It has been argued that, due to the scaling of the MaxEnt output, the number of background points used does not affect estimated probabilities (Elith et al., 2010)
- A study by (Phillips & Dudík, 2008) suggested that model performance reaches a plateau after 8,000 points. The majority of published studies have, consequently, adopted the MaxEnt default of using 10,000 points (e.g., Fourcade et al., 2014; Gormley et al., 2011; Wu et al., 2016)
- However, a study by Elith et al. (2011) suggested using different numbers of background points for regions that substantially differ in size, to ensure an equal representation of all environments
- In this study, preliminary trials were conducted to investigate the importance of the number of background points used
- Global models were developed using 10,000, 100,000, 200,000, 500,000 and 1M background points for England and Scotland
- Performance differences described by AUC scores were negligible between models. However, the response curves and rankings of variables by isolated training gain were more consistent between models using a minimum of 100,000 points
- As a result, the MaxEnt default of 10,000 background points was used for Wales only and 100,000 points were used for England and Scotland
- Increasing the number of background points in England and Scotland decreased the difference in densities of sampling points between the two regions (1.2 and 3.4 points per km^2^ for England and Scotland and Wales, respectively)

Environmental predictor variables

(1) Land cover

- Downloaded Centre of Ecology and Hydrology (CEH) Land Cover Map 2015 (25 x 25 m resolution, digimap.edina.ac.uk)
- Reclassified into 10 aggregate classes (Table 1)
- Resampled to 100 m resolution to match that of the species data (0.01 km^2^, majority value taken from 16 25 x 25m cells)
- Used the resampled land cover map as a base map to define the cell size and extent of all other layers (as this environmental dataset has the finest resolution)

(2) Land cover proportion

- Original data source used: CEH Land Cover Map 2015
- Calculated the cover of specific land cover types within an area
- Cover of each land type was calculated as a proportion (0-1) of the area within a 500 m radius buffer of each cell (0.785 km^2^ area) to include environmental information within an area that could reasonably be assumed to be occupied by an individual deer at the time of its observation
- Cover types were; (1) urban and suburban (low resources, poor cover, high disturbance - likely to be avoided by roe deer), (2) woodland (highest resources, highest canopy cover - most likely to be selected by roe deer) and (3) non-woodland forage (mostly grassland and arable, high resources for forage but low canopy cover and high disturbance – likely to be weakly favoured by roe deer)

(3) Roads

- Shapefiles for Motorways, A roads and B roads obtained the Meridian Transport Ordnance Survey map (available from digimap.edina.ac.uk)
- Road cover was calculated to differentiate between locations near to roads and those that are near to dense road networks (areas least likely to be occupied due to noise/light disturbance and mortality risk)
- As for percentage covers, calculated the cover of roads (m^2^) within a 500 m radius buffer of each cell
- Species data points within 100 m of a road removed from the model as it is likely that these originated from deer-vehicle collisions

(4) Terrain

- Downloaded Ordnance Survey Terrain 50 data (0.0025 km^2^ resolution, digimap.edina.ac.uk)
- Included elevation in the model as a surrogate for canopy cover in non-woodland habitat types. Although non-woodland forage was included as a land cover variable, habitat types within this category may vary in their level of canopy cover (providing shelter from detection and harsh weather), which is likely to influence habitat suitability for roe deer. Given the abundance of hedgerow trees and other tree features in lowland farm landscapes in Great Britain, it was assumed that elevation is negatively correlated with canopy cover in non-woodland habitat types and could therefore be used as a surrogate. It is recognised that such an assumption should be validated in future studies, for which LiDAR data would be suitable
- Used bilinear resampling to increase the cell size to 0.01 km^2^
- Produced additional layers for (1) Slope and (2) Rugosity ((Mean elevation within a 500 m radius buffer – elevation within 100 x 100 m cell) / elevation range within a 500 m radius buffer)

(5) Climate

- Downloaded data for 19 bioclimatic variables (30 arc secs resolution, $\sim$1 km^2^, worldclim.org, yearly mean values for 1970-2000)
- Used bilinear resampling to decrease the cell size to 0.01 km^2^ (NOTE: this step is necessary for the model but does not improve data resolution)
- The 19 variables were:

BIO1 = Annual Mean Temperature
BIO2 = Mean Diurnal Range (Mean of monthly (max. temp – min. temp))
BIO3 = Isothermality (BIO2/BIO7) (* 100)
BIO4 = Temperature Seasonality (standard deviation *100)
BIO5 = Max. Temperature of Warmest Month
BIO6 = Min. Temperature of Coldest Month
BIO7 = Temperature Annual Range (BIO5-BIO6)
BIO8 = Mean Temperature of Wettest Quarter
BIO9 = Mean Temperature of Driest Quarter
BIO10 = Mean Temperature of Warmest Quarter
BIO11 = Mean Temperature of Coldest Quarter
BIO12 = Annual Precipitation
BIO13 = Precipitation of Wettest Month
BIO14 = Precipitation of Driest Month
BIO15 = Precipitation Seasonality (Coefficient of Variation)
BIO16 = Precipitation of Wettest Quarter
BIO17 = Precipitation of Driest Quarter
BIO18 = Precipitation of Warmest Quarter
BIO19 = Precipitation of Coldest Quarter

(6) Omitted Data

- *Other deer species* - Resource use between different deer species is likely to be similar. A visual examination of the available species presence data for all six species in Great Britain and some exploratory analyses yielded no evidence to suggest that roe deer are displaced by any other species. As the available data are limited to presence-only observations, we chose to omit data for other deer species to reduce error from inconsistent sampling. It is unlikely that the populations of any of the six deer species in Great Britain are truly in equilibrium with their environment and the data show a strong spatial bias towards south-east England where many species were introduced/reintroduced
- *Hunter effort* – No consistent records are available for the entire study area. The National Gamebag Census data were considered but records for Wales are very limited.

### Variable selection

A priori selection

- From a candidate list of 33 variables, 22 were selected a priori (Table S1) based on existing knowledge of habitat preferences

Table S1. A priori selected environmental predictor variables used to develop the MaxEnt global model of roe deer (*Capreolus capreolus*) distribution in England and Scotland

| Variable class  (number of variables) | Predictor variable | Description | Units |
| --- | --- | --- | --- |
|  |  |  |  |
| **Land cover (7)** | Land cover | Land cover type within a 100 x 100 m cell | Categorical. 10 aggregated classes; (1) Broadleaved woodland; (2) Coniferous woodland; (3) Arable and horticulture; (4) Improved grassland; (5) Semi-natural grassland; (6) Mountain, heath & bog; (7) Saltwater; (8) Freshwater; (9) Coastal; (10) Urban and suburban |
|  | Woodland distance | Euclidean distance to the nearest broadleaved or coniferous woodland | m |
|  | Forage distance | Euclidean distance to the nearest non-woodland forage (Improved grassland, Semi-natural grassland or Arable land) | m |
|  | Urban distance | Euclidean distance to the nearest urban or suburban land | m |
|  | Woodland cover | Proportion of broadleaved and coniferous woodland within a 500 m radius buffer | 0-1 |
|  | Forage cover | Proportion of non-woodland forage (Improved grassland, Semi-natural grassland and Arable land) within a 500 m radius buffer | 0-1 |
|  | Urban cover | Proportion of urban and suburban land within a 500 m radius buffer | 0-1 |
| **Terrain (3)** | Elevation | Height above sea level | m |
|  | Rugosity | (Mean elevation within a 500 m radius buffer – elevation within 100 x 100 m cell) / elevation range within a 500 m radius buffer) | Standard deviation of elevation  (-1) - 1 |
|  | Slope | Maximum change in elevation over the distance between each 100 x 100 m cell and its eight neighbours | Degrees |
| **Climate (8)** | BIO1 | Annual Mean Temperature | °C x 10 |
|  | BIO6 | Min. Temperature of Coldest Month | °C x 10 |
|  | BIO7 | Temperature Annual Range (BIO5-BIO6) | °C x 10 |
|  | BIO11 | Mean Temperature of Coldest Quarter | °C x 10 |
|  | BIO12 | Annual Precipitation | mm |
|  | BIO13 | Precipitation of Wettest Month | mm |
|  | BIO15 | Precipitation Seasonality (Coefficient of Variation†) | mm |
|  | BIO16 | Precipitation of Wettest Quarter | mm |
| **Roads (4)** | Distance to Motorways | Euclidean distance to the nearest motorway | m |
|  | Distance to A roads | Euclidean distance to the nearest A road | m |
|  | Distance to B roads | Euclidean distance to the nearest B road | m |
|  | Road cover | Area of motorways, A roads and B roads within a 500 m radius buffer | m^2^ |
|  |  |  |  |
|  |  |  |  |

- †Calculated as the ratio of the standard deviation to the mean of annual precipitation ranges for each year from 1960 to 1990

Checking for collinearity

- Although MaxEnt is less sensitive to high collinearity than traditional statistical models (Elith et al., 2011), it is recommended that highly correlated predictors are removed to aid interpretation (Dormann et al., 2013; Merow et al., 2013)
- Pairwise Pearson’s correlation coefficients were calculated and correlation plots were created in R (R Core Development Team, 2019)using data extracted from a random background sample of 100,000 points (Table S2)
- Any highly correlated ($\left| r \right|\geq0.7$) variables within the same variable class (Table S1) were identified for potential removal
- Correlation between variables of different classes was not considered cause for variable removal as the model remained easily interpretable without any exclusions (as expected, several climatic variables were highly correlated with elevation)

Removing highly correlated variables

- A MaxEnt model was developed using all a priori selected variables (Table S1),

100,000 random background points and presence data for England in Scotland divided 10-fold into a training dataset (n = 383) and test dataset (n = 3460), hereafter referred to as the global model

- Variable importance was assessed using a jackknife test to observe the regularized training gain produced by each variable when used in isolation (Gormley et al., 2011)
- If two variables within the same variable class (Table S1) were correlated, the variable that produced the lowest regularized training gain when used in isolation was removed
- A set of 14 variables were retained (Table S3) and used to develop a second model using 100,000 random background points and presence data for England and Scotland divided 10-fold into a training dataset (n = 383) and test dataset (n = 3460), hereafter referred to as the trial model

Table S2. Pairwise Pearson’s correlation coefficients calculated for environmental predictor variables in the global MaxEnt habitat suitability model for roe deer (*Capreolus capreolus*) in England and Scotland. Variables retained for the trial model are highlighted in bold text.

Reducing model complexity

- Optimal model complexity was determined based on observations of the corrected Akaike information criterion (AICc) in a similar stepwise approach to that described by (Zeng et al., 2016)
- Environmental predictor variables in the trial model were ranked in order of variable importance (Table S3)
- A model was developed using the full variable set and the AICc was calculated from MaxEnt’s raw output using the ENMeval package in R (Muscarella et al., 2014)
- The lowest ranking variable was then removed and the AICc calculation was performed again
- The stepwise removal of variables continued until only two variables remained (Zeng et al., 2016)
- The model with the lowest AICc (Table S4) was selected for tuning and is hereafter referred to as the selected model

Table S3. Environmental predictor variables retained for the trial MaxEnt habitat suitability model for roe deer (*Capreolus capreolus*) distribution in England and Scotland following collinearity analysis. Variables are ranked in order of importance as described by the regularized training gain produced by each variable when used in isolation.

| Predictor variable | Isolated training gain | Rank |
| --- | --- | --- |
|  |  |  |
| Woodland distance | 0.202 | 1 |
| Land cover | 0.175 | 2 |
| Woodland cover | 0.153 | 3 |
| Forage distance | 0.085 | 4 |
| Forage cover | 0.082 | 5 |
| Urban distance | 0.062 | 6 |
| BIO1 (Annual mean temperature) | 0.055 | 7 |
| Elevation | 0.049 | 8 |
| BIO15 (Precipitation seasonality) | 0.026 | 9 |
| Road cover | 0.022 | 10 |
| Urban cover | 0.016 | 11 |
| Rugosity | 0.006 | 12 |
| BIO7 (Temperature annual range) | 0.004 | 13 |
| Slope | 0.002 | 14 |
|  |  |  |

Table S4. Akaike Information Criterion (AICc) values for the MaxEnt habitat suitability models for roe deer (*Capreolus capreolus*) distribution in England and Scotland following stepwise variable removal. Each AICc value assigned to a predictor variable is derived from an independent model developed using that variable and all higher-ranking variables.

| Variable rank | Predictor variable | AICc | Model rank |
| --- | --- | --- | --- |
|  |  |  |  |
| 1 | Woodland distance |  |  |
| 2 | Land cover | 12696.7 | 3 |
| 3 | Woodland cover | 12729.4 | 7 |
| 4 | Forage distance | 12714.8 | 6 |
| 5 | Forage cover | 12696.9 | 4 |
| 6 | Urban distance | 12689.1 | 1 |
| 7 | BIO1 (Annual mean temperature) | 12706.6 | 5 |
| 8 | Elevation | 12730.1 | 8 |
| 9 | BIO15 (Precipitation seasonality) | 12689.3 | 2 |
| 10 | Road cover | 12735.8 | 9 |
| 11 | Urban cover | 12745.0 | 11 |
| 12 | Rugosity | 12737.7 | 10 |
| 13 | BIO7 (Temperature annual range) | 12788.4 | 12 |
| 14 | Slope | 12867.9 | 13 |
|  |  |  |  |

Model tuning

- Tuning of the selected model was performed with all 48 possible combinations of the following feature classes: linear (L), linear+quadratic (LQ), hinge (H), linear+quadratic+hinge (LQH), linear+quadratic+hinge+product (LQHP) and linear+quadratic+hinge+product+threshold (LQHPT) and eight regularization coefficients (from 0.5 to 4 with increments of 0.5, for more information on model tuning and feature selection see Phillips & Dudík 2008)
- Model performance was assessed based on AICc values
- The combination of parameters that achieved the lowest AICc value was used for the evaluated model (Table S5)

Table S5. Parameter settings of the best performing model (with the lowest Akaike Information Criterion, AICc) following model tuning including feature classes (L= Linear, Q= Quadratic, H= Hinge, P= Product) and regularization coefficient (β)

| Predictor variables | Feature class | β |
| --- | --- | --- |
|  |  |  |
| Woodland distance, Land cover, Woodland cover, Forage distance, Forage cover, Urban distance | L,Q,H,P | 1.0 |
|  |  |  |

**S2. RangeShifter model**

### Cost surface maps

**
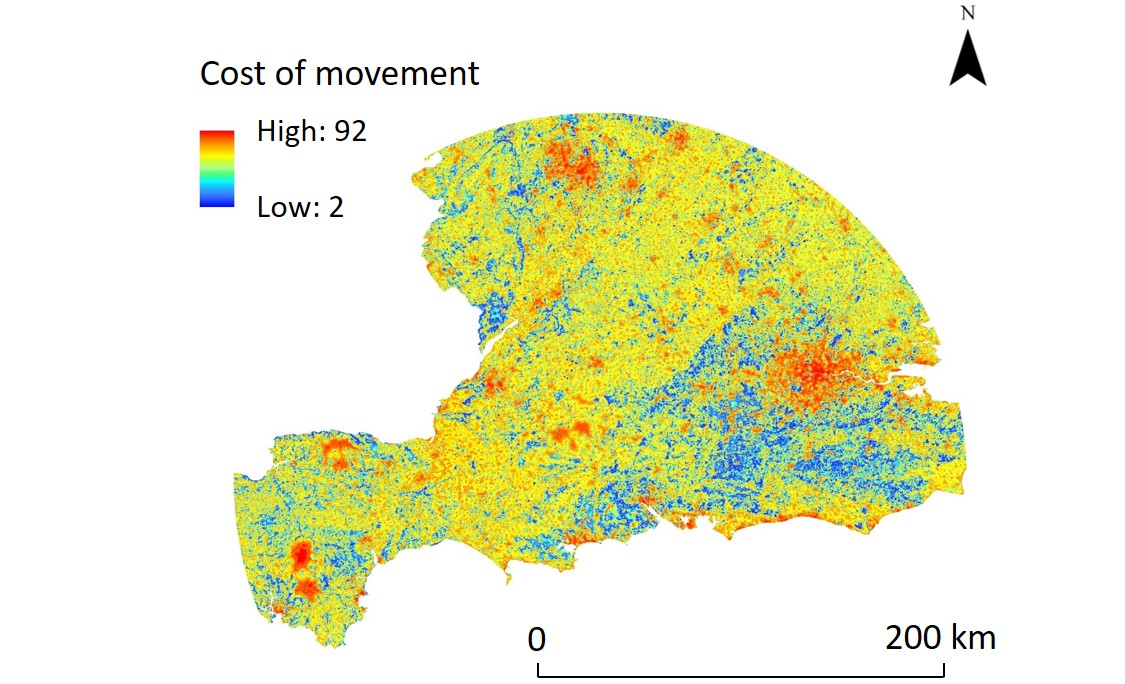
**

Figure S1. Cost surface of the Historic Area of Expansion (HAE) for roe deer in southern England derived from the MaxEnt habitat suitability model. Costs are scaled from 0 (no resistance) to 100 (completely impermeable to movement).


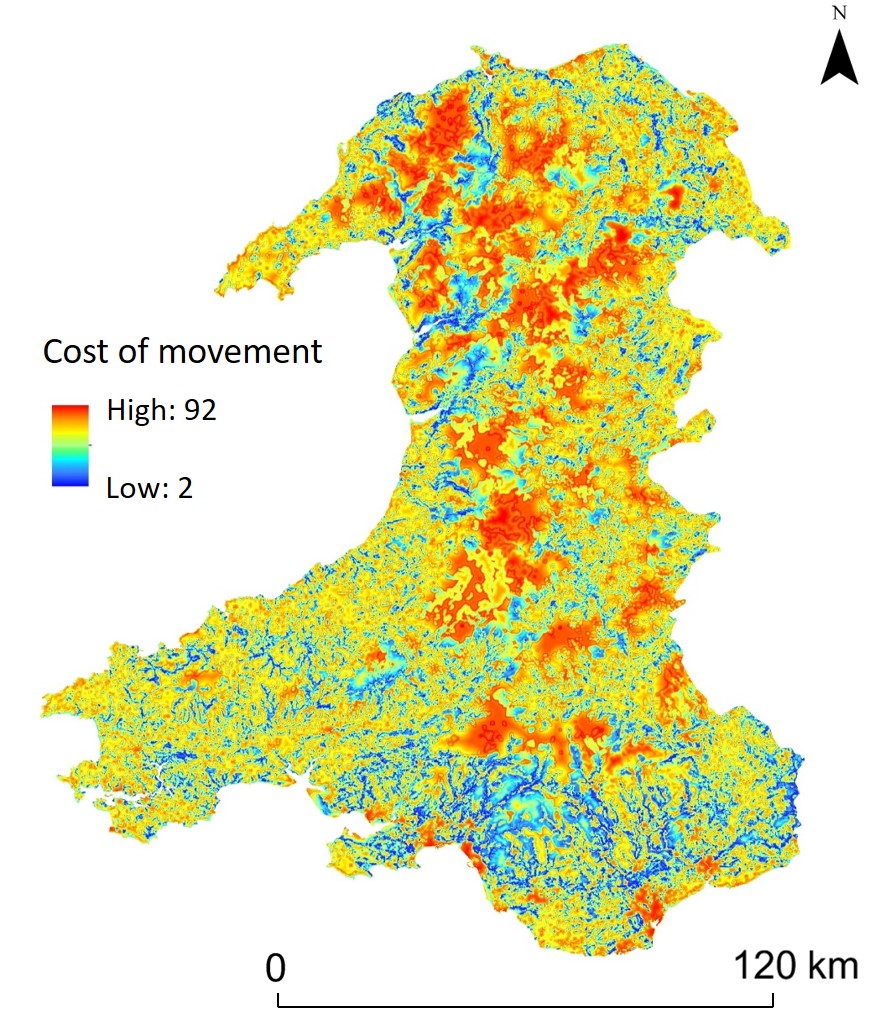


Figure S2. Cost surface of Wales for roe deer derived from the MaxEnt habitat suitability model. Costs are scaled from 0 (no resistance) to 100 (completely impermeable to movement).

### Initialization parameters

Historic Area of Expansion

Simulations were initialized with the species occupying all patches within a 10 km radius buffer of the geographic centre of the 1960-1969 range. The centre of the observed range was calculated using GIS by extracting the centroid of the minimum convex polygon at ObsTS1 into a point feature. Patches were occupied at carrying capacity and the distribution of ages was at quasi-equilibrium. Each simulation was run for 300 years, which was estimated to be sufficient to achieve complete occupation of all available patches in each model based on preliminary trials. Patch occupancy was estimated at regular 50-year intervals, corresponding to six simulated timesteps (SimTS1 to SimTS6). Mean patch occupancy was estimated from a total of 10 simulations.

Wales

Simulations were initialised with the species occupying patches within 10 x 10 km grid-cells that intersect observations of species presence (Fig. 3). Patches were occupied at carrying capacity and the distribution of ages was at quasi-equilibrium. Each simulation was run for 150 years, which was estimated to be sufficient to achieve complete occupation of all available patches based on preliminary trials. Patch occupancy was estimated at regular 15-year intervals, corresponding to ten simulated timesteps (SimTS1 to SimTS10). Mean patch occupancy was estimated from a total of 10 simulations.

### Sensitivity analysis

Table S6. Results of the sensitivity analysis showing the spatial agreement (TSS) between simulations from the Voronoi-Contiguity (Vor-Con) model and the observed species range (ObsRange) and the distribution of presences within observed ranges (ObsPresences) for roe deer in the HAE from 1960-2016.

|  |  |  | TSS† | | | | |
| --- | --- | --- | --- | --- | --- | --- | --- |
| Observation | Parameter | Setting | Mean | SD | Min | Med | Max |
|  |  |  |  |  |  |  |  |
|  |  |  |  |  |  |  |  |
| ObsRange | Directional Persistence | 3 | 0.68 | 0.17 | 0.44 | 0.72 | 0.87 |
|  |  | 7 | 0.67 | 0.19 | 0.37 | 0.72 | 0.87 |
|  | Maximum number of steps | 180 | 0.68 | 0.17 | 0.44 | 0.72 | 0.87 |
|  |  | 220 | 0.68 | 0.17 | 0.44 | 0.72 | 0.87 |
|  | Perceptual Range | 2 | 0.69 | 0.17 | 0.49 | 0.73 | 0.85 |
|  |  | 6 | 0.67 | 0.17 | 0.42 | 0.71 | 0.86 |
|  | **Vor-Con model** | ***** | **0.69** | **0.16** | **0.44** | **0.72** | **0.86** |
|  |  |  |  |  |  |  |  |
|  |  |  |  |  |  |  |  |
|  |  |  |  |  |  |  |  |
| ObsPresences | Directional Persistence | 3 | 0.52 | 0.12 | 0.39 | 0.51 | 0.67 |
|  |  | 7 | 0.52 | 0.12 | 0.40 | 0.51 | 0.67 |
|  | Maximum number of steps | 180 | 0.52 | 0.12 | 0.39 | 0.51 | 0.67 |
|  |  | 220 | 0.52 | 0.12 | 0.39 | 0.51 | 0.67 |
|  | Perceptual Range | 2 | 0.54 | 0.16 | 0.37 | 0.51 | 0.75 |
|  |  | 6 | 0.54 | 0.15 | 0.38 | 0.53 | 0.72 |
|  | **Vor-Con model** | ***** | **0.53** | **0.16** | **0.37** | **0.52** | **0.74** |
|  |  |  |  |  |  |  |  |

TSS: True skill statistic, HAE: Historic Area of Expansion, * parameters: Directional Persistence = 5, Maximum number of steps = 200, Perceptual range = 4; † values calculated from six simulated time steps.

### Model evaluation

The following figures illustrate characteristics of the model evaluation procedure. In each figure we show how the predicted range from the hybrid model was compared to the observed range (ObsRange, estimated by constructing minimum convex polygons around presences, *MCP range*) and presence locations within the observed range (ObsPresences). The results (right-most panel and table) are comprised of correctly predicted presences/absences (‘True positives’/‘True negatives’, respectively) and incorrectly predicted presences/absences (‘False positives’/‘False negatives’, respectively). The number of cells assigned to each category determines model sensitivity (i.e., the proportion of predicted presences that were correct = True positives $\div$ (True positives + False positives)), specificity (i.e., the proportion of predicted absences that were correct = True negatives $\div$ (True negatives + False negatives)) and the True Skill Statistic (TSS) = (sensitivity + specificity) – 1. Examples are given for hypothetical models that (A) performed well and (B) performed poorly at early and late timesteps (Fig. S3 and S4, respectively).


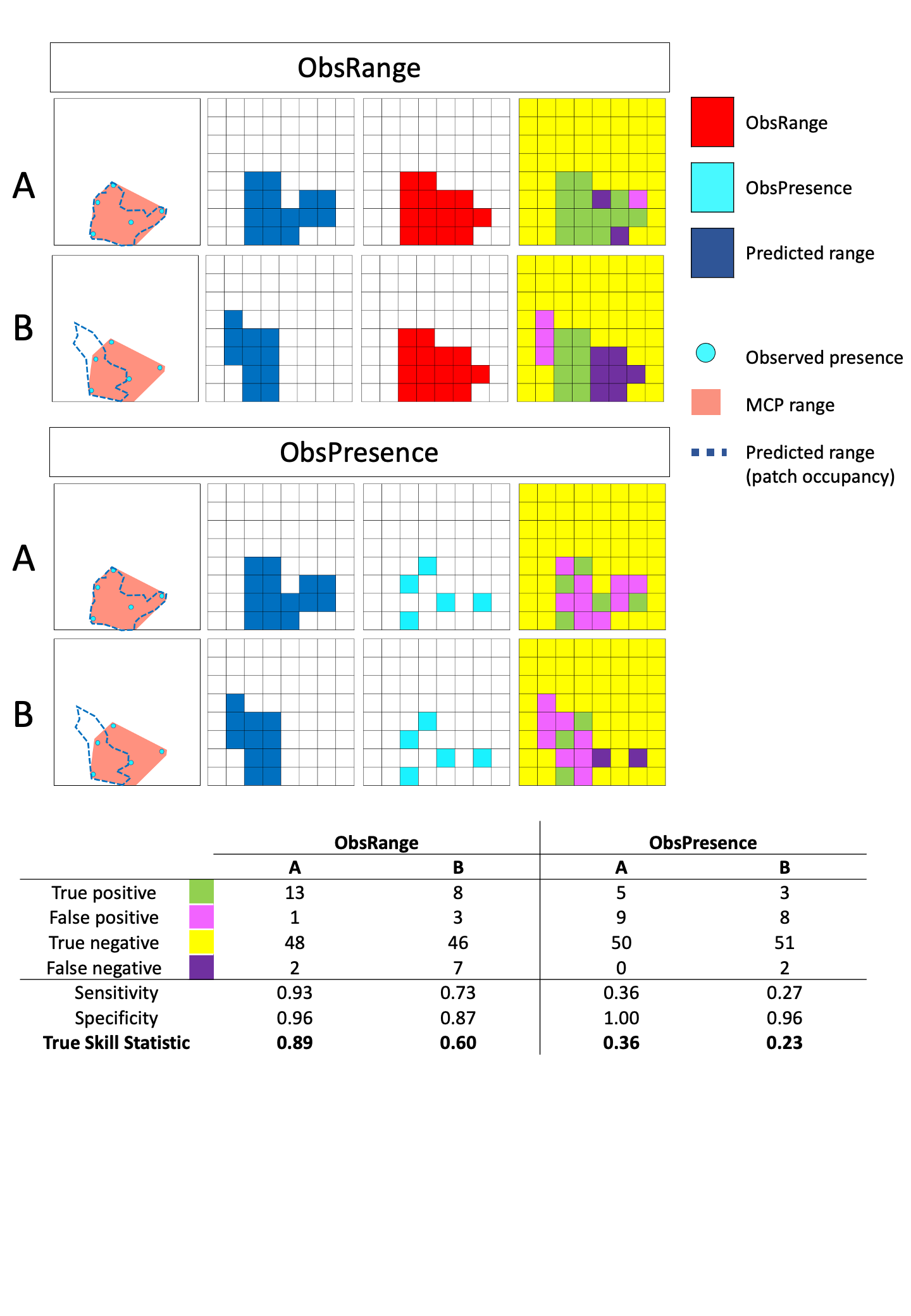


Figure S3. Illustration of model evaluation results at an early timestep for hypothetical models that (A) performed well and (B) performed poorly (see text for key definitions).


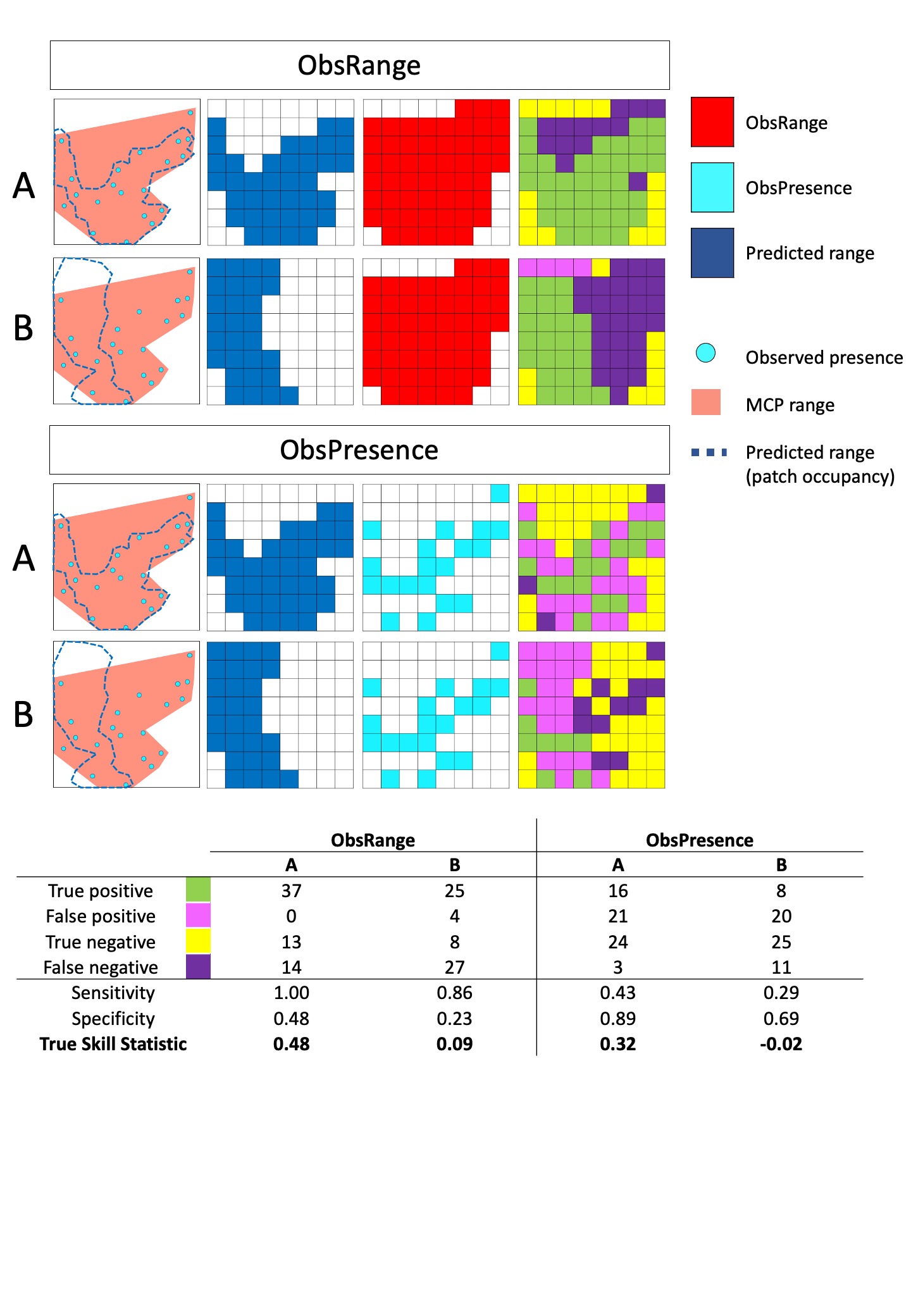


Figure S4. Illustration of model evaluation results at a late timestep for hypothetical models that (A) performed well and (B) performed poorly (see text for key definitions).

Table S7. Evaluation results for the RangeShifter (Bocedi, Palmer, et al., 2014) hybrid model showing the spatial agreement (True Skill Statistic, *TSS = (sensitivity + specificity )- 1*, Allouche et al., 2006), between the simulated range and the observed species range (ObsRange) at six timesteps (ObsTS).

| Generalisation method | ObsTS | Sensitivity | Specificity | TSS |
| --- | --- | --- | --- | --- |
|  |  |  |  |  |
| Grid | 1 | 1.00 | 0.83 | 0.83 |
|  | 2 | 1.00 | 0.88 | 0.88 |
|  | 3 | 0.98 | 0.85 | 0.82 |
|  | 4 | 0.90 | 0.78 | 0.69 |
|  | 5 | 0.76 | 0.67 | 0.43 |
|  | 6 | 0.93 | 0.34 | 0.27 |
|  |  |  |  |  |
| Voronoi | 1 | 1.00 | 0.83 | 0.83 |
|  | 2 | 1.00 | 0.88 | 0.88 |
|  | 3 | 0.98 | 0.82 | 0.79 |
|  | 4 | 0.93 | 0.72 | 0.65 |
|  | 5 | 0.79 | 0.76 | 0.54 |
|  | 6 | 0.93 | 0.41 | 0.35 |
|  |  |  |  |  |
| Contiguity | 1 | 1.00 | 0.84 | 0.84 |
|  | 2 | 0.55 | 0.95 | 0.50 |
|  | 3 | 0.57 | 0.95 | 0.52 |
|  | 4 | 0.64 | 0.86 | 0.50 |
|  | 5 | 0.73 | 0.83 | 0.56 |
|  | 6 | 0.92 | 0.50 | 0.42 |
|  |  |  |  |  |
| Vor-Con | 1 | 1.00 | 0.83 | 0.83 |
|  | 2 | 1.00 | 0.87 | 0.87 |
|  | 3 | 0.99 | 0.84 | 0.82 |
|  | 4 | 0.95 | 0.72 | 0.67 |
|  | 5 | 0.85 | 0.85 | 0.71 |
|  | 6 | 0.93 | 0.65 | 0.58 |
|  |  |  |  |  |

Table S8. Evaluation results for the RangeShifter (Bocedi, Palmer, et al., 2014) hybrid model showing the spatial agreement (True Skill Statistic, *TSS = (sensitivity + specificity )- 1*, Allouche et al., 2006), between the simulated range and the observed presences (ObsPresences) at six timesteps (ObsTS).

| Generalisation method | ObsTS | Sensitivity | Specificity | TSS |
| --- | --- | --- | --- | --- |
|  |  |  |  |  |
| Grid | 1 | 1.00 | 0.86 | 0.86 |
|  | 2 | 0.86 | 0.92 | 0.79 |
|  | 3 | 0.77 | 0.94 | 0.71 |
|  | 4 | 0.54 | 0.94 | 0.48 |
|  | 5 | 0.41 | 0.90 | 0.32 |
|  | 6 | 0.43 | 0.85 | 0.28 |
|  |  |  |  |  |
| Voronoi | 1 | 1.00 | 0.86 | 0.86 |
|  | 2 | 0.84 | 0.92 | 0.76 |
|  | 3 | 0.78 | 0.92 | 0.69 |
|  | 4 | 0.58 | 0.92 | 0.50 |
|  | 5 | 0.42 | 0.91 | 0.34 |
|  | 6 | 0.43 | 0.85 | 0.28 |
|  |  |  |  |  |
| Contiguity | 1 | 0.89 | 0.87 | 0.77 |
|  | 2 | 0.45 | 0.98 | 0.43 |
|  | 3 | 0.40 | 0.98 | 0.39 |
|  | 4 | 0.35 | 1.00 | 0.35 |
|  | 5 | 0.39 | 1.00 | 0.39 |
|  | 6 | 0.42 | 0.93 | 0.35 |
|  |  |  |  |  |
| Vor-Con | 1 | 1.00 | 0.86 | 0.86 |
|  | 2 | 0.83 | 0.90 | 0.74 |
|  | 3 | 0.77 | 0.93 | 0.70 |
|  | 4 | 0.61 | 0.93 | 0.54 |
|  | 5 | 0.46 | 0.95 | 0.40 |
|  | 6 | 0.42 | 0.95 | 0.37 |
|  |  |  |  |  |

REFERENCES:

Dormann, C. F., Elith, J., Bacher, S., Buchmann, C., Carl, G., Carré, G., Marquéz, J. R. G., Gruber, B., Lafourcade, B., Leitão, P. J., Münkemüller, T., Mcclean, C., Osborne, P. E., Reineking, B., Schröder, B., Skidmore, A. K., Zurell, D., & Lautenbach, S. (2013). Collinearity: A review of methods to deal with it and a simulation study evaluating their performance. Ecography, 36(1), 27–46. https://doi.org/10.1111/j.1600-0587.2012.07348.x

Elith, J., Phillips, S. J., Hastie, T., Dudík, M., Chee, Y. E., & Yates, C. J. (2011). A statistical explanation of MaxEnt for ecologists. Diversity and Distributions, 17(1), 43–57. https://doi.org/10.1111/j.1472-4642.2010.00725.x

Fourcade, Y., Engler, J. O., Rödder, D., & Secondi, J. (2014). Mapping species distributions with MAXENT using a geographically biased sample of presence data: A performance assessment of methods for correcting sampling bias. PLoS One, 9(5), e97122. https://doi.org/10.1371/journal.pone.0097122

Gormley, A. M., Forsyth, D. M., Griffioen, P., Lindeman, M., Ramsey, D. S. L., Scroggie, M. P., & Woodford, L. (2011). Using presence‐only and presence‐absence data to estimate the current and potential distributions of established invasive species. Journal of Applied Ecology, 48(1), 25–34. https://doi.org/10.1111/j.1365-2664.2010.01911.x

Merow, C., Smith, M. J., & Silander, J. A. (2013). A practical guide to MaxEnt for modeling species' distributions: What it does, and why inputs and settings matter. Ecography, 36(10), 1058–1069. https://doi.org/10.1111/j.1600-0587.2013.07872.x

Muscarella, R., Galante, P. J., Soley‐Guardia, M., Boria, R. A., Kass, J. M., Uriarte, M., & Anderson, R. P. (2014). ENMeval: An R package for conducting spatially independent evaluations and estimating optimal model complexity for Maxent ecological niche models. Methods in Ecology and Evolution, 5(11), 1198–1205. https://doi.org/10.1111/2041-210x.12261

Phillips, S. J., Dudi'k, M., Dudi'k, D., Elith, J., Graham, C. H., Lehmann, A., Leathwick, J., & Ferrier, S. (2009). Sample selection bias and presence‐only distribution models: Implications for background and pseudo‐absence data. Ecological Applications, 19(1), 181–197.

Wu, W., Li, Y., & Hu, Y. (2016). Simulation of potential habitat overlap between red deer (Cervus elaphus) and roe deer (Capreolus capreolus) in northeastern China. PeerJ, 2016(3), e1756. https://doi.org/10.7717/peerj.1756

Zeng, Y., Low, B. W., & Yeo, D. C. J. (2016). Novel methods to select environmental variables in MaxEnt: A case study using invasive crayfish. Ecological Modelling, 341, 5–13. https://doi.org/10.1016/j.ecolmodel.2016.09.019
